# Protein Modifications for Cellular Protein Delivery

**DOI:** 10.1021/acs.chemrev.6c00267

**Published:** 2026-08-13

**Authors:** Jan Vincent V. Arafiles, Jonathan Franke, Palina Dubatouka, Luise Franz, Christian P.R. Hackenberger

**Affiliations:** † 28417 Leibniz-Forschungsinstitut für Molekulare Pharmakologie (FMP), Robert-Rössle-Straße 10, 13125 Berlin, Germany; ‡ Department of Chemistry, Humboldt-Universität zu Berlin, Brook-Taylor-Straße 2, 12489 Berlin, Germany

## Abstract

Intracellular delivery of functional proteins is emerging as a powerful strategy to interrogate and control cellular pathways with high spatial and temporal precision. Recent advances in chemical biology now enable the design of cell-permeable proteins through precise chemical and biochemical modification, bringing the field closer to achieving intracellularly targeted biologics as a “new class of drugs”. By overcoming the limitations of genetic manipulation, researchers create solutions for basic research and medicine. In this review, we outline the key motivations that drive intracellular protein delivery. We highlight central mechanistic paradigms for protein entry, important parameters influencing protein delivery, and summarize analytical tools to assess successful delivery. We outline different chemical and biochemical conceptional approaches that resulted in breakthrough studies to achieve functional protein transport. Finally, we discuss ongoing efforts, highlighting the challenges for future research on protein delivery.

## Graphical abstract

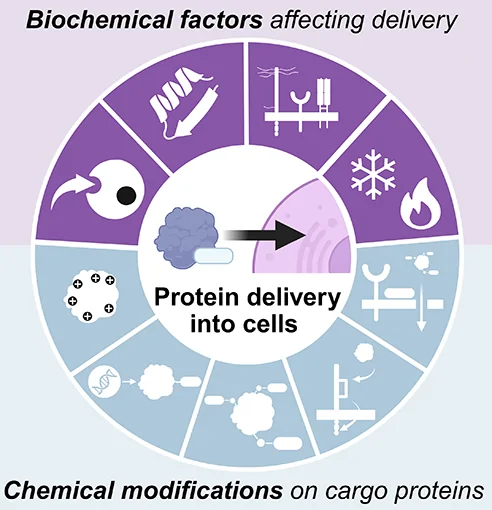

## 1 Introduction

*“How can I make a molecule cell-permeable?”* While this question has both fascinated and despaired generation of researchers, it certainly led to numerous concepts, rules and theories to understand and improve cell permeability, especially for small molecules.^1,2^ Having access to different cell delivery modalities should address one of the central challenges in molecular life science and pharmaceutical sciences: understanding and regulating intracellular function. Given the fact that proteins are the main components in orchestrating physiological function, and that they are usually not cell permeable, it may not come as a surprise that a particularly important question in current research in chemical biology is “*How can I make a protein cell-permeable?”*

Numerous breakthroughs in genetic engineering and protein biochemistry have provided various avenues for studing and manipulating cellular functions by producing, degrading, or modulating protein expression.^3–8^ Although these molecular biology-based alterations have greatly advanced our understanding of cellular physiology, these techniques often disregard the effects of genetic manipulation on cells and fail to delineate the vast complexity of the cell’s proteome. In recent years, chemical biology groups have provided numerous chemistry-driven enabling technologies that can probe fundamental biological questions neglected by standard biochemical approaches. Technologies such as bioorthogonal reactions,^9–11^ proximity labeling,^12^ chemoselective peptide ligations and modifications,^13–16^ semisynthetic protein synthesis strategies,^17^ or the expression of proteins with noncanonical amino acids,^18–20^ provided chemists, biochemists, and biologists with innovative tools to expand and challenge the fundamentals of cell biology.

These advancements were, in our opinion, also vital to breakthroughs in the field of intracellular delivery of proteins. Impressive achievements in functional protein synthesis helped researchers visualize the prospect of delivering specifically modified and functionally equipped proteins into cells, while chemoselective modification reactions are instrumental in creating cell-permeable proteins by connecting them to appropriate delivery vectors with unprecedented precision. Other approaches, including physical or mechanical methods, polymers, nanoparticles, and viral and bacterial nanocarriers, are already covered in other excellent reviews.^21–25^ Therefore, this Review focuses on **how chemical and biochemical protein modification strategies enable and shape the cellular delivery of proteins** (Figure 1).

**Figure 1:**
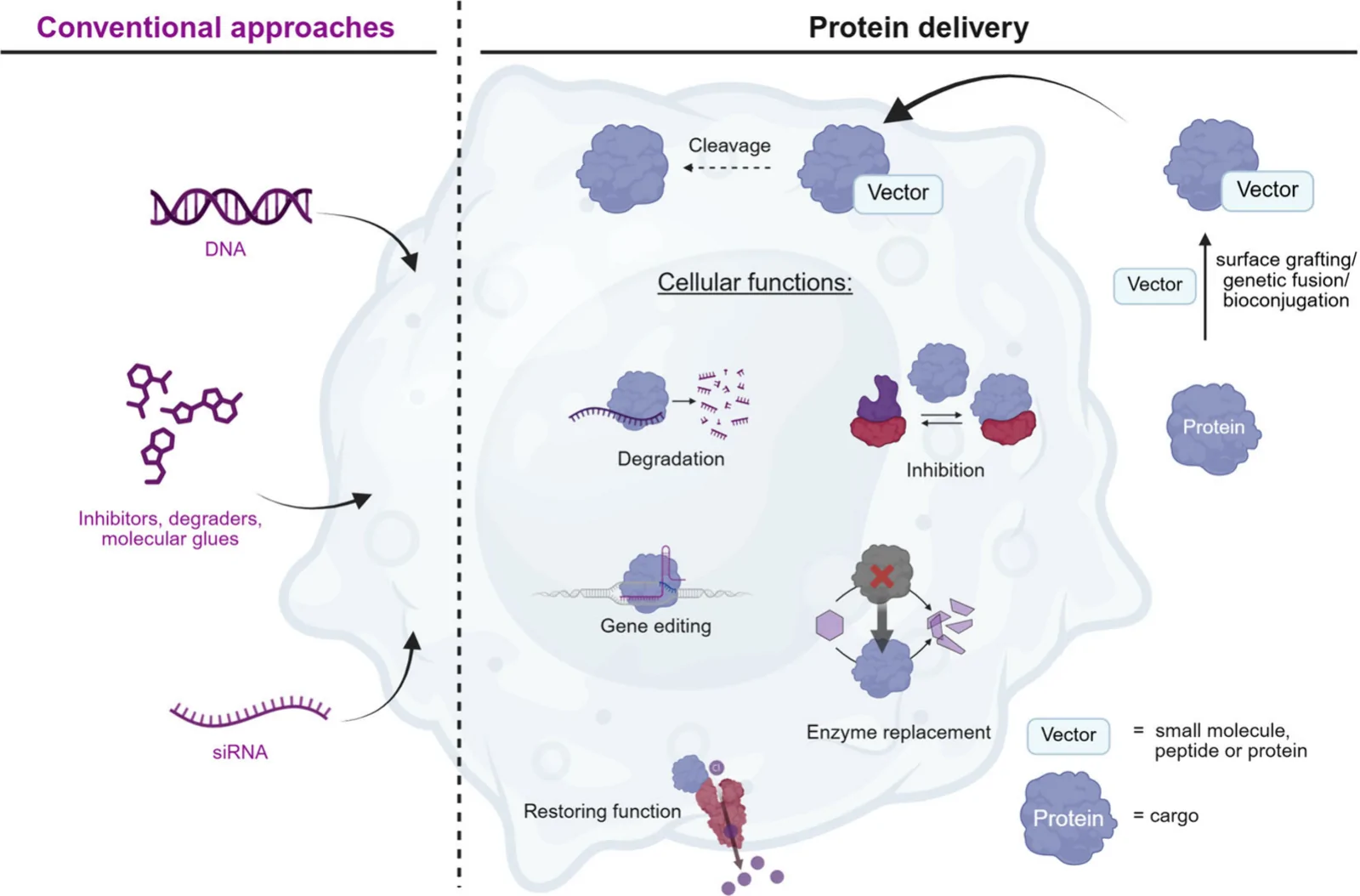
Protein modifications for cellular delivery as alternative to conventional approaches. Highlighted are some potential applications for manipulation and interrogation of intracellular functions. Created with https://BioRender.com.

We begin by illustrating the main research goals and motivations for basic and translational research, which fundamentally drive intracellular protein delivery strategies in the next section. Afterward, we provide an overview of the central mechanistic paradigms for cellular entry pathways of proteins, namely, energy-dependent endosomal uptake versus the energy-independent route that directly bypasses the cellular membrane, commonly termed protein translocation in section 3 . We then highlight recurring parameters that are reported to determine successful delivery of various protein-based cargoes, including common and sophisticated analytical techniques for quantification. Section 4 presents different conceptual approaches that enable cellular delivery by modifying proteins, using either biochemical or chemical approaches. Finally, we discuss ongoing efforts to advance the field and raise questions and concerns that are relevant to future research in protein delivery.

## 2 Why Do We Need Protein Delivery?

By enabling access to proteins carrying functional modules or specific post-translational modifications using state-of-the-art protein (semi)­synthesis techniques, it is possible to precisely probe or tune intracellular pathways without genetic manipulation. This section highlights the prospects and discusses the key influence of protein delivery in basic research and pharmaceutical applications.

### 2.1 Basic Research

Cytosolic and nuclear delivery of exogenous wild-type or chemoenzymatically modified proteins allows direct interrogation of intracellular pathways, while providing the precise temporal control needed to uncover molecular mechanisms (Figure 2).^21,24,26,27^ Since no genetic alterations are performed, protein delivery offers a possibility to peek into the cell’s physiology in the natural setting.^21^ Using the protein delivery methods presented in this review, researchers have made highly valuable contributions to basic research.

**Figure 2:**
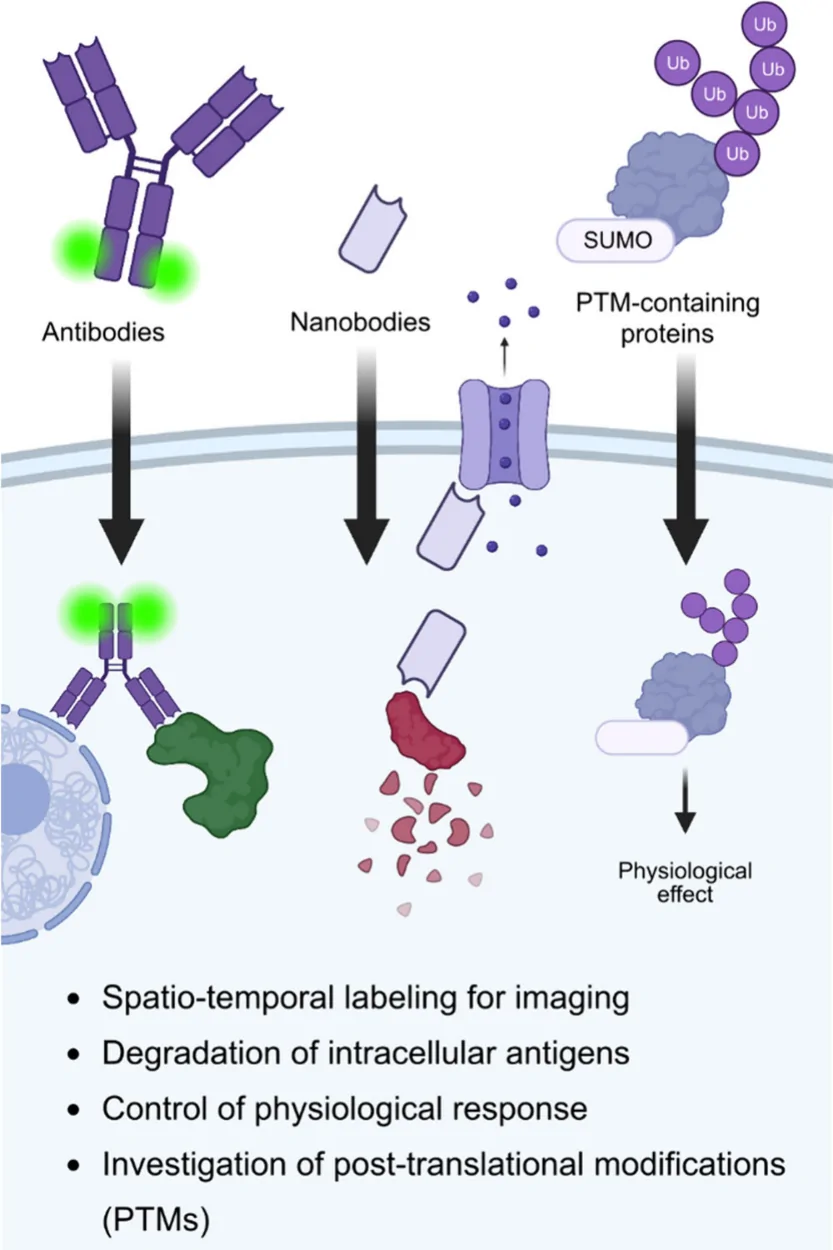
Selected applications of protein delivery for basic research. Created with https://BioRender.com.

Among many research directions, a major line of interest is devoted to providing cell-permeable antibody formats.^28–33^ Full-length antibodies are routinely used in a multitude of biochemical assays; nevertheless, their lack of cell permeability in live cells prevents scientists from using them to their fullest capacity.^33^ In recent years, many groups across all disciplines in chemical biology, and even computational design, have worked toward creating means for antibody delivery.^34–37^ Moreover, recent trends in biochemistry place particular emphasis on camelid-derived single-chain nanobodies, which are easily expressed and chemically modified. After Cardoso, Hackenberger, and co-workers demonstrated that delivered cell-permeable nanobodies can bind to intracellular antigens.^29^ Several groups used these modalities for applications within living cells, including the visualization of endogenous intracellular targets using dual-color super-resolution microscopy^32^ (Figure 3), chemically induced proximity and ferroptosis induction,^38^ specific degradation of protein targets,^39^ and inhibition of active opioid receptors.^40^ Most recently, the Hackenberger group reported the delivery of functional nanobodies to elicit an intracellular response, even in primary cells from cystic fibrosis (CF) patients. They engineered a cell-permeable nanobody that intracellularly corrects and stabilizes the F508del CF transmembrane conductance regulator (CFTR), restoring its chloride channel function and further improving the performance of the clinically approved drug CF-drug TRIKAFTA^(R)^.^41^

**Figure 3:**
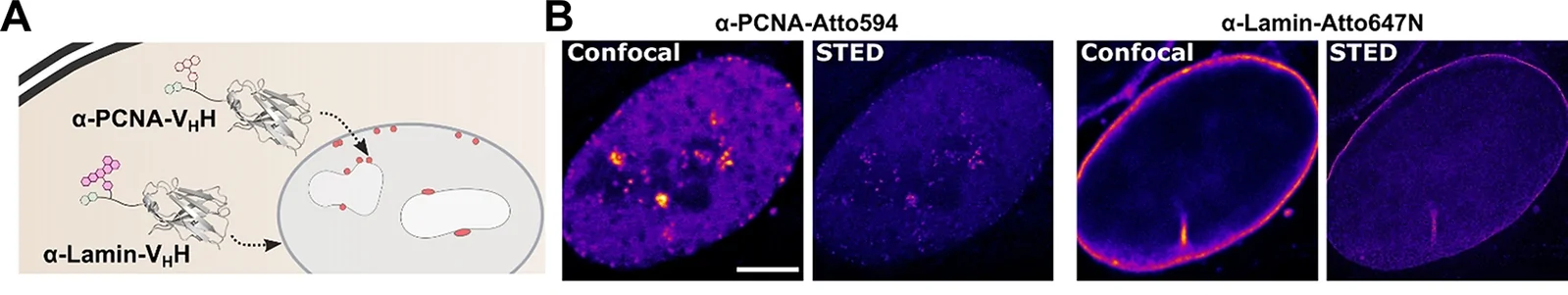
Representative application of cell-permeable nanobodies for super-resolution stimulated emission depletion (STED) microscopy. Reproduced with permission from ref 32. Copyright 2021 John Wiley and Sons.

Studying post-translational modifications (PTMs) is another key area in chemical biology that increasingly benefits from extracellular protein delivery.^42^ Precise investigations of PTMs are challenging, especially given their cellular heterogeneity and the obstacles to probing specific PTMs using genetic methods.^43^ Building upon elaborate chemical strategies to synthesize modified proteins with specific biologically relevant chemical modifications,^17,44,45^ researchers are becoming increasingly interested in interrogating the physiological roles of specific PTMs.^46–49^ Highlights include the development of cell-permeable activity-based ubiquitin probes to profile deubiquitinases by Zhuang and co-workers^50^ and the chemical synthesis of cell-permeable ubiquitin and small ubiquitin-like modifiers (SUMO) by the Brik group to visualize phosphorylated ubiquitin^48^ as well as ubiquitinylation and SUMOylation^51^ events in live cells (Figure 4). In addition, PTM-modified proteins were delivered for *in situ* mapping of PTM-mediated interactions by Liu and Li et al.^46^ We could expect that with increasing capacities and modalities for protein delivery, more studies will utilize chemically synthesized, PTM-containing cell-permeable proteins to study the physiological roles of specific PTMs.

**Figure 4:**
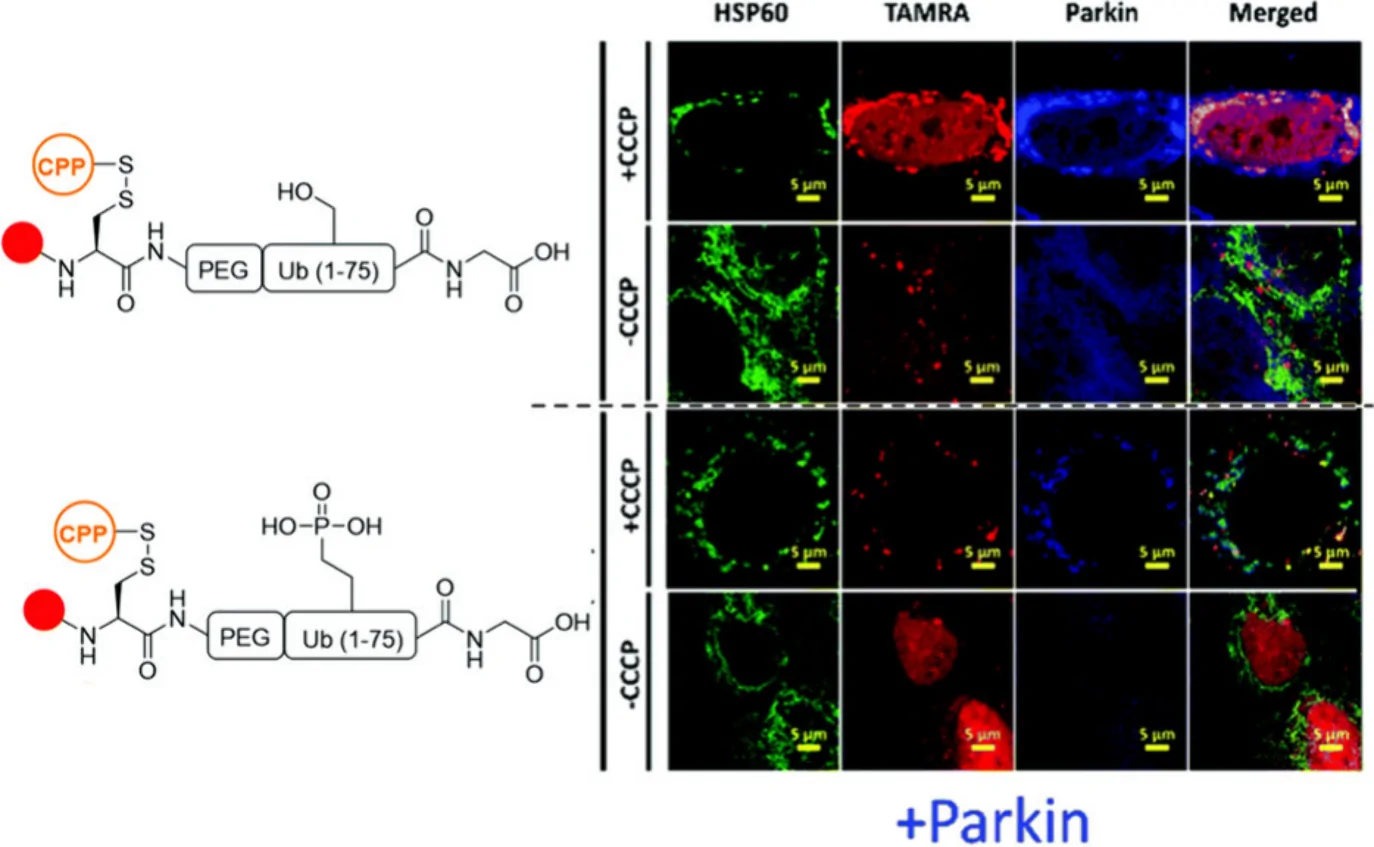
Cellular delivery and colocalization of phosphorylated ubiquitin with Parkin protein. Adapted with permission from ref 48. Copyright 2021 Royal Society of Chemistry.

### 2.2 Biopharmaceuticals

Biopharmaceuticals have continued to be highly successful in recent years, accounting for roughly 30% of newly FDA-approved drugs in 2023–2025. While monoclonal and bispecific antibodies dominate this field, it should be noted that most approved protein therapeutics target soluble ligands, extracellular matrix components, or surface receptors.^21,52^ Nevertheless, many of the most attractive disease drivers, including mutant RAS,^53^ MYC,^54^ intracellular kinases, transcription factors,^55^ and viral replication complexes^56^ reside in the cytosol or nucleus and are often “undruggable” by small molecules. Direct intracellular delivery of often non-permeable peptide and protein modalities such as peptide macrocycles, enzymes, inhibitory scaffolds, antibodies and CRISPR ribonucleoproteins, would allow highly selective modulation of these targets, with rapid onset and transient action.^57–59^ Unlocking this space is a major goal in chemical biology and pharmaceutical research; still, the gap between experimental delivery tools and approved drugs remains wide. A review by Chan and Tsourkas provides a comprehensive comparison of protein-based therapeutic modalities versus small molecules, while also highlighting the conditions under which protein-based drugs are most beneficial.^21^

There are a few notable approved protein drugs that achieve intracellular action.^60,61^ Prominent examples include enzyme replacement therapies (ERTs) for lysosomal storage diseases (Figure 5). Recombinant lysosomal enzymes (e.g., imiglucerase, agalsidase, or alglucosidase alfa) are engineered to present mannose-6-phosphate (M6P) or other mannose residues, which are recognized by M6P-receptors on the surface of target cells. These enzymes are internalized and trafficked along the endolysosomal pathway to lysosomes, where they degrade accumulated substrates and reverse storage pathology.^62^ Dedicated reviews on ERTs for the treatment of lysosomal storage diseases are available.^60,63,64^

**Figure 5:**
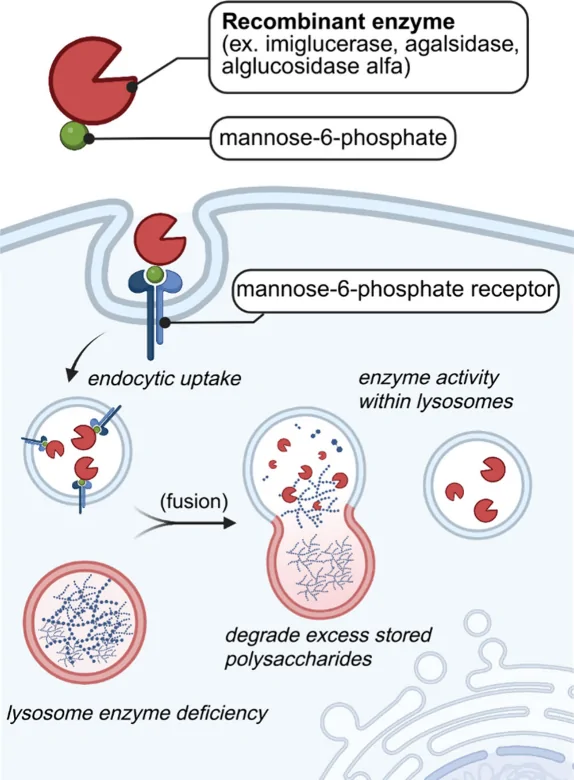
Schematic diagram of enzyme replacement therapy (ERT). ERT uptake does not require endosomal release since the activities of these enzymes are expected in the lysosomes. Created with https://BioRender.com.

The therapeutic efficacy and dose requirements for ERT are highly dependent on how efficiently the exogenous enzyme is routed to lysosomes rather than exerting their effects within the cytosol.^65–67^ An example of a cytosol-oriented protein therapeutic is denileukin diftitox (Ontak), which is a fusion of interleukin-2 (IL-2) with a truncated diphtheria toxin.^68,69^ Upon binding to IL-2-receptors on T-cell malignancies, the fusion protein is internalized and the diphtheria toxin fragment translocates to the cytosol, where it inactivates the elongation factor-2 and shuts down protein synthesis.^70^ FDA-approved Immunotoxins, such as moxetumomab pasudotox (Lumoxiti), are other examples of protein therapeutics that require intracellular delivery. Moxetumomab pasudotox is an anti-CD22 recombinant antibody fused to a fragment of Pseudomonas exotoxin A (PE38).^71^ This immunotoxin is internalized after binding to CD22 on B-cell leukemias, where PE38 is released into the cytosol and ribosylates the elongation factor-2 to halt protein synthesis, leading to apoptotic cell death.^72^ While these two examples demonstrate that protein toxins can indeed be delivered to cytosolic targets in patients through antibody conjugation, their clinical use is still limited by narrow therapeutic windows and significant toxicities.^68,69,71,73^

IDAXI/Daxxify was approved by the FDA in 2022 to treat moderate to severe glabellar lines associated with hyperactive contractions of the corrugator supercilii and procerus muscle, representing the first approved therapeutic protein-based modality containing a cell-penetrating peptide (CPP) as a delivery agent.^74^ The active component, a 150 kDa botulinum neurotoxin type A protein, is formulated together with RTP004, a 35-amino acid peptide, which consists of a lysine backbone flanked by two terminal cationic CPP sequences. This highly positively charged peptide adopts a polyproline type II helix conformation in solution and binds noncovalently to the anionic surfaces of the neurotoxin, preventing surface adsorption and aggregation while increasing presynaptic binding and cellular uptake.^75^

Beyond these examples, there are no approved protein-based drugs designed to bind a cytosolic or nuclear target as their primary pharmacological mechanism, a point emphasized in recent reviews.^76^ This has led researchers to consider intracellularly targeted biologics an aspirational “new class of drugs”, especially as the experimental toolkit for intracellular delivery continues to expand.

## 3 What Must Be Considered When Delivering Proteins?

The main limitations to effective and efficient intracellular delivery of proteins are cell-biological and biophysical in nature.^77^ First, the plasma membrane, which evolved to be highly efficient at keeping unneeded materials, chemicals, and organisms out while allowing only very specific, cellularly necessary components into the cytoplasm, is effectively impermeable to exogenous proteins. Proteins are large, highly polar, and often charged; therefore, they neither diffuse across lipid bilayers nor use endogenous transporters.This impermeability is even more apparent in plant cells due to the presence of the glycan cell wall.^78^ In the interest of length, this Review will focus on mammalian cell delivery. This section discusses the main biochemical and biophysical mechanisms that allow intracellular delivery of extracellular proteins (section 3.1), emerging parameters that govern uptake (section 3.2), and appropriate analytical methods to characterize and quantify protein uptake (section 3.3).

### 3.1 Entry Pathways

For most large, hydrophilic protein-based materials and therapeutics, entry through the plasma membrane is considered one of the biggest challenges. Largely due to the membrane’s tight regulation, extracellular proteins generally cannot cross without either initiating a physiological response (i.e., endocytosis) through interactions with cell surface proteins or eliciting physicochemical changes that enable direct translocation. This section will briefly discuss the two main routes of entry into the cell and the challenges associated with each (Figure 6).

**Figure 6:**
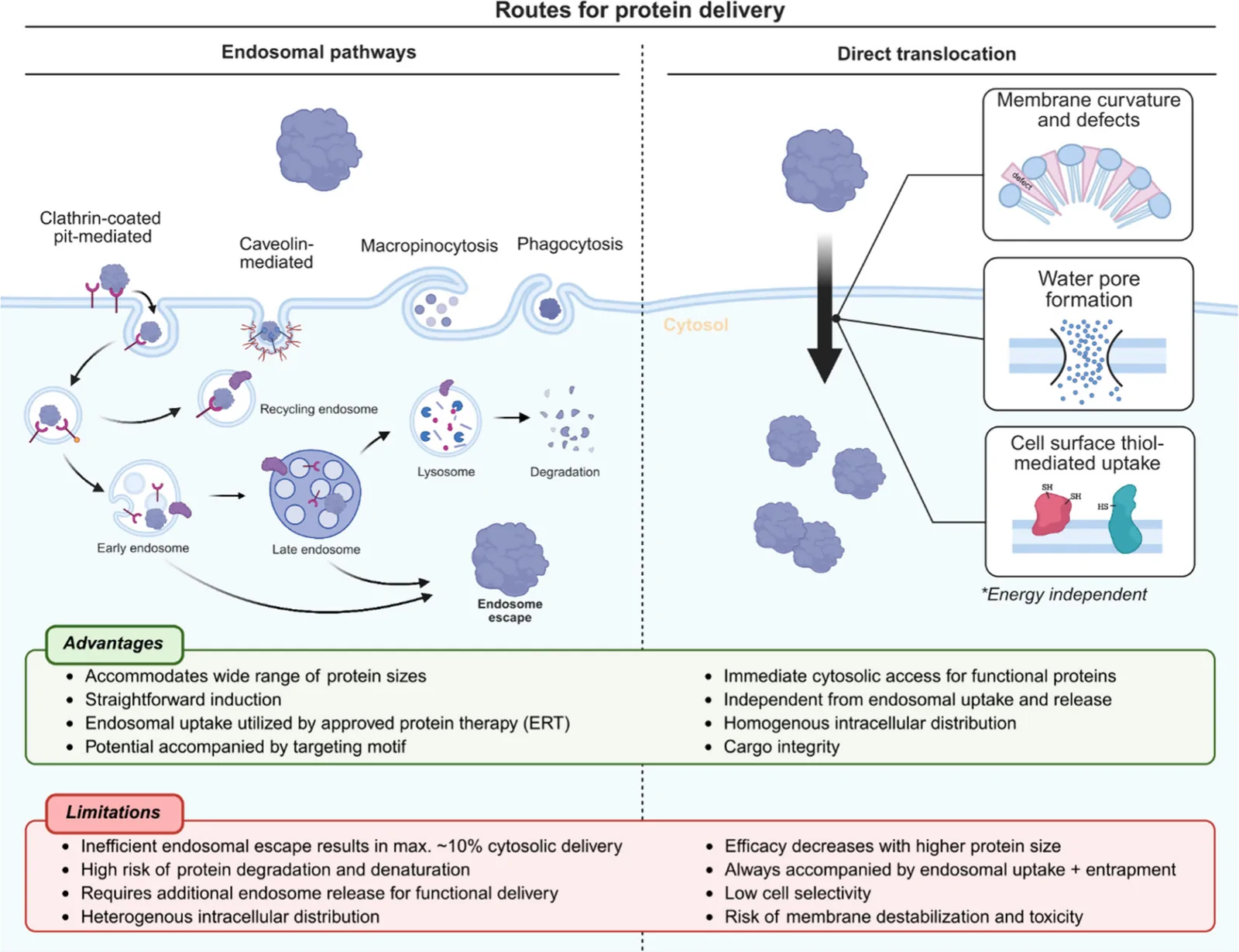
Biochemical pathways for protein delivery with their respective advantages and limitations. Endosomal pathways are diverse but always require endosomal escape for cytosolic delivery of protein. Direct translocation ensures immediate cytosolic availability and proceeds through membrane deformation. Created with https://BioRender.com.

#### 3.1.1 Endocytosis

Efficient endocytic uptake is initiated by the association of delivery vehicles and protein cargoes with specific cell-surface components and receptors.^79^ Endocytic pathways are heavily reliant on ATP/GTP-dependent cellular machineries (e.g., coat protein association, and membrane scission by dynamin) and are regulated by phosphorylation and signaling events. Therefore, these processes are largely energy-dependent.^80–83^ Since the biomolecular interplay involved in receptor-mediated endocytosis is well documented, this review will focus on endosomal entry routes for protein cargoes and refer readers to dedicated reviews on the biological basis of endocytosis.^79^ (Figure 6)

Once activated, the mechanistic route of protein cargo internalization is dictated by a complex interplay among physiological responses.^79^ Physicochemical properties, such as protein surface charge, size, protein folding, extracellular concentration, and the specific target cell type, all affect the specific endosomal pathway that is activated. Clathrin-mediated endocytosis, caveolae/lipid-raft-dependent endocytosis, and macropinocytosis are reported as the most common endocytic routes activated by protein delivery strategies.

Clathrin-mediated endocytosis (CME) is the most well-characterized pathway, involving the formation of clathrin-coated vesicles that undergo scission from the plasma membrane and are typically around 100 nm in diameter.^84,85^ CME is initiated by the clustering of endocytic proteins at the cell membrane by receptor–ligand interactions. This event is followed by a cascade of biological events from the recruitment of adapter proteins (e.g., BAR domain proteins), assembly of clathrin coat proteins, membrane bending, and membrane scission.^85^ While CME is the primary route for many nutrient ligands, its restricted vesicle size can limit the types of macromolecular cargoes it can effectively transport. In particular, enzymes used in enzyme replacement therapies (ERT, see section 2.2) enter cells via CME and are transported to lysosomal compartments, where their therapeutic activity is exerted.^86–89^ Groups also found that cargoes exert a direct physical effect on membrane invagination and on the entire endocytosis progression and maturation.^90,91^ Strategies such as receptor-targeted protein conjugates and CPP-conjugated proteins are commonly reported to pass through CME, however, this uptake pathway is not beneficial for proteins intended for the cytosol due to endosome recycling and entrapment, which will be discussed later in section 3.1.3. For a more thorough discussion about the biological processes involved in CME, the authors refer the readers to other works.^84,92^

Caveolae/lipid raft-dependent endocytosis is also reported to facilitate the delivery of macromolecules into cells.^93,94^ Similar to CME, this endocytic pathway requires an initial activation through receptor–ligand interaction, such as the activation of receptor tyrosine kinases. This process, along with the accumulation of cargoes on lipid rafts and flask-shaped plasma membrane invaginations, termed caveolae, initiates the uptake mechanism. This pathway utilizes hydrophobic, cholesterol-rich microdomains and are often exploited by certain viruses and small proteins to avoid immediate lysosomal degradation.^95^ Interestingly, peptides such as the sweet arrow peptide (SAP) from the *N*-terminus of the maize storage protein γ-zein, human calcitonin-derived peptide hCT(9–32), and transportan have been reported to induce this endocytic pathway.^96–98^ These peptides are amphiphilic and exhibit a high degree of membrane interaction compared with CME-inducing vectors.^98–101^

Macropinocytosis is also a commonly attributed endocytic pathway used by various delivery strategies.^102–106^ Often characterized as a “bulk” fluid-phase endocytic process, macropinocytosis is an actin-driven pathway used by cell for nutrient scavenging.^107^ It is frequently triggered by the interaction of cationic proteins or arginine-rich CPPs with the cell surface, specifically the glycocalyx layer. This interaction leads to signaling events such as Rac1 activation and subsequent actin reorganization.^108–110^ Noncationic peptides, such as the flock house virus-derived peptide,^111^ SDF-1α-derived SN21,^112^ and P4A peptides and their analogs^113,114^ were also reported to induce macropinocytosis via the activation of cell-surface receptors.

#### 3.1.2 Direct Translocation

Direct translocation, also known as transduction or direct membrane penetration, is a non-endocytic pathway by which macromolecules traverse the plasma membrane lipid bilayer to reach the cytoplasm.^23^ Unlike endocytosis, it allows the delivery of proteins directly through the plasma membrane, resulting in immediate bioavailability within the cytosol.This process is generally considered energy-independent. Although direct translocation is independent of cellular metabolic machinery, it is often driven by electrostatic interactions, membrane potential, and macromolecular self-assembly, all of which are not exempt from physical free-energy cost (Figure 7).^115,116^ Methods that can be categorized as direct translocation include mechanophysical methods, which rely on lipid membrane destabilization/disruption through electroporation,^117,118^ microinjection,^119^ mechanoporation,^120^ or cell squeezing,^121^ and contractile bacterial injection systems.^122–124^

**Figure 7:**
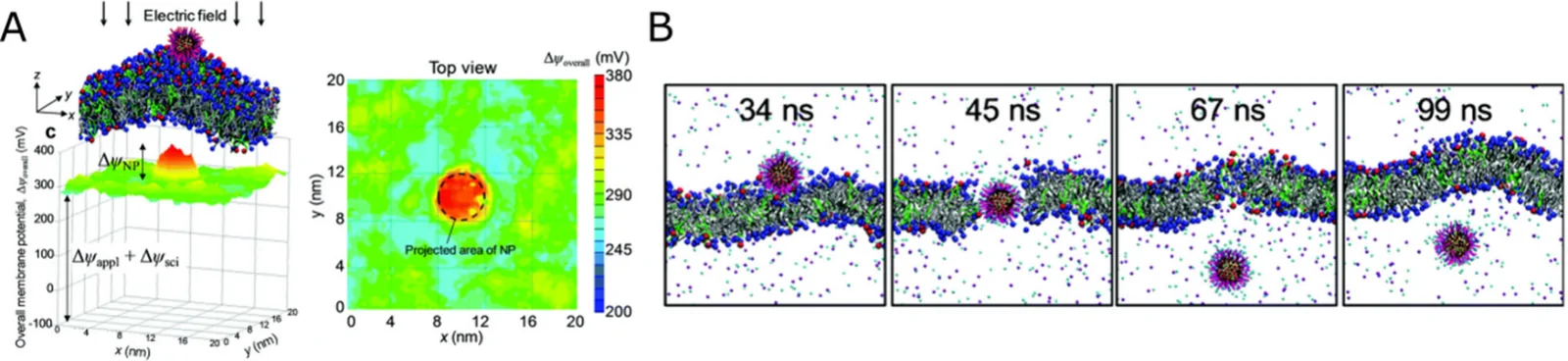
MD simulation of direct translocation of cationic nanoparticles through the cell membrane. Reproduced with permission from ref 116. Copyright 2019 Royal Society of Chemistry.

Direct translocation has also been exploited by proteins to perform signaling and regulatory functions across cellular boundaries. The archetypal example is the HIV-1 transactivator of transcription (TAT) protein, which can rapidly translocate across the plasma membrane and accumulate in the cell nucleus.^125,126^ The cell penetration ability of this protein was largely attributed to a short sequence of the cationic peptide, from which the first cell-penetrating peptide, “TAT” (sequence: GRKKRR­QRRRPPQ) was deduced.^125–127^ Another example is the VP22 peptide (sequence: NAKTRR­HERRRK­LAIER) derived from the VP22 protein of the herpes simplex virus, a DNA-binding protein that demonstrated rapid, energy-independent nuclear accumulation.^128^ Homeoproteins are transcription factors, such as the *Drosophila* Antennapedia protein, that possess unique paracrine properties, allowing them to be secreted by one cell and internalized into another via their third helical domain from which the CPP “penetratin” (sequence: RQIKIW­FQNRRM­KWKK) was constructed.^129^ Several reviews address the diversity of CPPs capable of performing direct translocation.^130–133^ Recent advances in protein delivery have expanded the possibilities for direct cellular translocation by employing site-specific bioconjugation strategies of CPPs, which will be discussed in section 4.3. While once debated as an experimental artifact, current evidence confirms direct translocation as a distinct entry route for both natural proteins and engineered protein conjugates. Recent developments in the field by different groups contributed further mechanistic understanding required to elucidate entry routes for protein–vector conjugates.^115,134–137^

#### 3.1.3 Endosome Entrapment and Escape

While endosomal uptake is often highly efficient and offers the prospect of cell-specific delivery via receptor targeting, internalized protein cargoes must exit the endosomes (i.e., endosomal escape) to be therapeutically effective.^138,139^ Once internalized, the vast majority of protein cargoes follow a canonical trafficking route from early to late endosomes and finally to lysosomes.^140^ During this maturation process, the vesicles undergo progressive acidification (from pH ∼6.5 to ∼4.5) and acquire various proteolytic enzymes, jeopardizing protein cargos. Researchers attempted to provide different quantitative frameworks to determine how many endocytosed proteins are released into the cytosol, revealing a massive disparity between efficient uptake and functional delivery.^141^ Summarizing key literature, even with endosomal escape vectors, less than 10% of endocytosed cargos successfully reach the cytosol, while over 90% remain trapped and are highly likely degraded in endosomes, a phenomenon commonly referred to as the “endosomal bottleneck”.^141–143^ Consequently, researchers have focused on developing “endosomolytic” modules to disrupt the vesicle membrane and liberate the cargo. Well-received examples include the pH-responsive peptides GALA^144^ or pH-Low Insertion Peptide (pHILIP),^145^ as well as lipid-sensitive peptides, such as attenuated cationic amphiphilic peptides^146^ or dfTAT,^147^ which undergo conformational switches in lysosomal or endosomal environments.^148^ Given the large number of successful strategies reported over the years, we refer interested readers to focused reviews on endosome release strategies.^22,149^

Direct translocation is advantageous over endocytosis uptake by overcoming the endosomal bottleneck. Naturally, non-endosomal uptake pathways preserve cargo integrity by avoiding exposure to the acidic, protease-rich endolysosomal environment, which can compromise protein stability and function. The rapid uptake of protein cargo into the cytosol provides functional proteins with immediate access into the cytosol. It is important to note that endosomal and non-endosomal uptake are not binary processes but rather exist on a spectrum influenced by experimental conditions.^103^ A protein-vector may enter via direct translocation at high concentrations (typically >10 μM) while relying entirely on endocytosis at low therapeutically relevant concentrations.^150^ A good example of such a concentration-dependent vector are polyarginine CPPs. Achieving exclusive direct translocation is challenging since the requisite membrane interaction process invariably triggers concomitant endosomal uptake.^100,151^ However, in the absence of specific design features for endosomal escape, protein conjugates remain trapped within endosomes.^152^ Furthermore, as cargo size increases from as small as peptides (1–2 kDa) to full-length antibodies (160 kDa), the likelihood of direct translocation decreases, and the reliance on endocytosis increases proportionally with size.^153^

### 3.2 Emerging Parameters Influencing Uptake

Following the different routes of protein entry into cells, we further elaborate on the factors that influence the efficiency and efficacy of the protein delivery methods. This section will focus on the physicochemical properties of cargo proteins and delivery vectors, various cell surface entities, and microenvironmental states that affect the efficiency and efficacy of protein delivery (Figure 8).

#### 3.2.1 Protein Cargos and Delivery Vectors

When designing a cell-permeable protein, it is necessary to consider the properties of the protein cargo and the delivery vector. Moreover, careful assessment of potential functional consequences upon cargo/vector conjugation and protein-to-vector compatibility should be undertaken.

**Figure 8:**
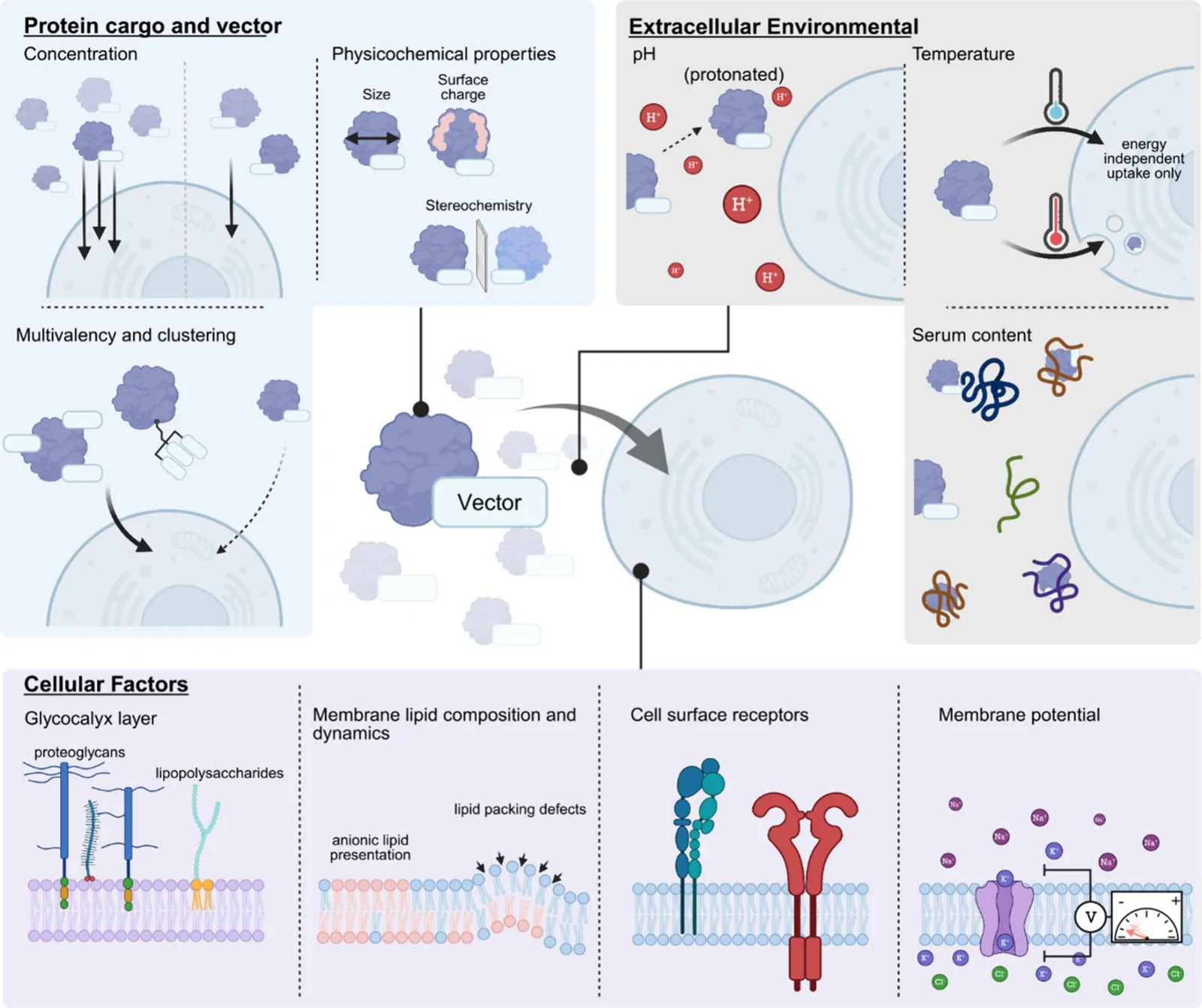
Key emerging parameters that govern the uptake of chemically modified proteins. Created with https://BioRender.com.

##### 3.2.1.1 Protein Cargo Size

Physicochemical properties, such as size, polarity, and surface net charge, significantly influence the delivery success of a given protein, regardless of the choice of delivery vector. The protein size is a crucial determinant of delivery success (Figure 9).^153^ A report by Mier et al. highlights this by showing the challenge of reaching high-efficiency delivery of large proteins, such as antibodies or Cas9, whereas smaller proteins are generally less problematic.^154^ Additionally, different uptake pathways (endosomal or direct translocation) have limitations regarding the cargo size that they can accommodate.^79,134,137^ This is quite evident for CPP-mediated direct translocation through water pores, where the size of the water pores formed, determines the size of cargo entering the cell.^134,137^ This size threshold of specific pathways might limit the kinds of proteins that each pathway accommodates; hence, careful investigations must be performed to choose the best uptake pathway for protein cargoes of a specific size.^155^

**Figure 9:**
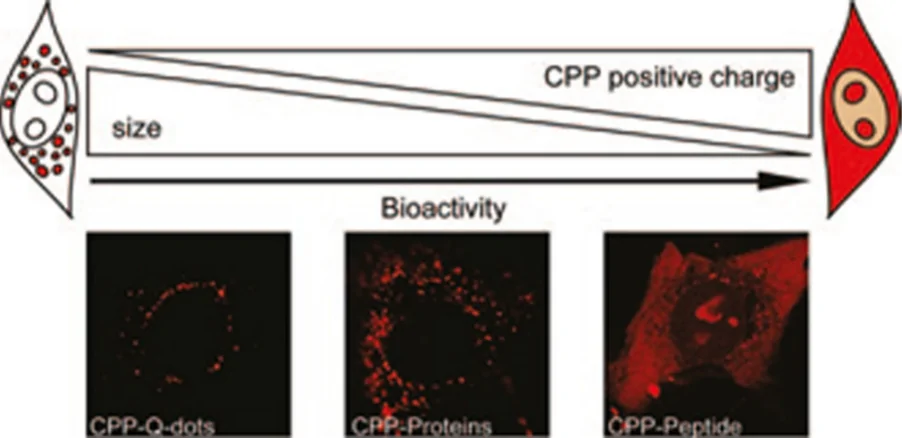
Effect of protein cargo size and the number of positive charges on overall protein delivery. Reproduced with permission from ref 153. Copyright 2006 John Wiley and Sons.

##### 3.2.1.2 Protein Cargo Surface Charge and Isoelectric Point

Protein cargo surface charge was independently demonstrated and reported by the Raines and Liu groups to significantly impact delivery performance (discussed in section 4.1). Permeability generally increases with increasing positive charge and masking negatively charged amino acids.^156^ Moreover, inherently positively charged proteins, such as Cre recombinases, are easily delivered into cells. Recent reports also showed that proteins having a higher number of arginine peptide modifications on the protein surface showed more efficient protein delivery into cells.^157^

Similarly, the isoelectric point (pI) of the protein of interest must also be considered. pI refers to the pH at which a protein carries a net charge of zero. Proteins typically exhibit minimal solubility near their pI and may therefore precipitate under such conditions;^158^ hence, a chosen delivery vector may influence the pI of its cargo protein, affecting its stability and deliverability. For example, anionic modification could decrease the pI of the protein of interest, thus making the cargo more tolerant to acidic conditions.^159^ Yao and co-workers fused a superpositive transduction domain (K4), with a theoretical charge of +40, to proteins with pI values ranging from 3.7 to 10.6.^160^ The addition of such a highly cationic motif to proteins shifts the apparent pI of the fusion protein toward the basic range and helps maintain a net positive charge at physiological pH, improving the overall cellular delivery of the proteins. Methods that influence the protein surface charge, and to some degree the pI, of protein cargoes will be discussed in section 4.1 of this review.

##### 3.2.1.3 Protein Cargo Chirality

Recently, the impact of chirality was investigated by Brik and co-workers in response to the growing interest in mirror-image proteins for biopharmaceutical research.^14,161,162^ The group found that d-protein cargoes showed decreased delivery efficiency compared with their natural l-counterparts, mainly due to differential engagement of energy-dependent endocytic pathways, whereas chirality had no effect on passive translocation. A similar observation was reported by Pellois and co-workers, who observed reduced endocytic uptake of d-amino acid TAT peptides.^163^ With increasing efforts in mirror-image protein synthesis, we can expect that groups investigating d-life will eventually tackle deeper questions regarding how chirality influences delivery efficiency.^161^

##### 3.2.1.4 Delivery Vector Properties

Likewise, delivery vectors greatly influence uptake and delivery, which will be further discussed in section 4. A recurring concept to increase the efficiency of protein delivery is the introduction of multivalency into the vector.^164^ Multiple groups have used clustering or multimerization of delivery vectors to increase delivery efficiency and to transport challenging protein cargoes, such as antibodies. Pellois and co-workers used dimerized D-TAT,^165^ while Vallis and co-workers employed tricyclic TAT to promote endosomal escape of native IgG antibodies.^36^ The association of trimeric TAT with the cargo promoted the delivery via reversible attachment of an anionic peptide patch on the protein.^46^ Futaki and co-workers observed an interesting phenomenon in which trimerized endosomolytic, amphiphilic L17E peptide resulted in the formation of liquid droplets or coacervates that efficiently delivered antibodies via a combination of energy-dependent actin reorganization and membrane ruffling (Figure 10).^166^ Similarly, Hackenberger and co-workers introduced the CPP-additive technology, which relies on cell surface accumulation and clustering to deliver CPP-conjugated protein cargo.^34^ This technology will be further discussed in section 4.3.^34,137,167^ While presented in separate subsections, one should not overlook the possibility that cargo and vector molecules may form unproductive interactions upon conjugated.^168^ Therefore, the compatibility of the cargo protein and the delivery vector requires careful consideration.

**Figure 10:**
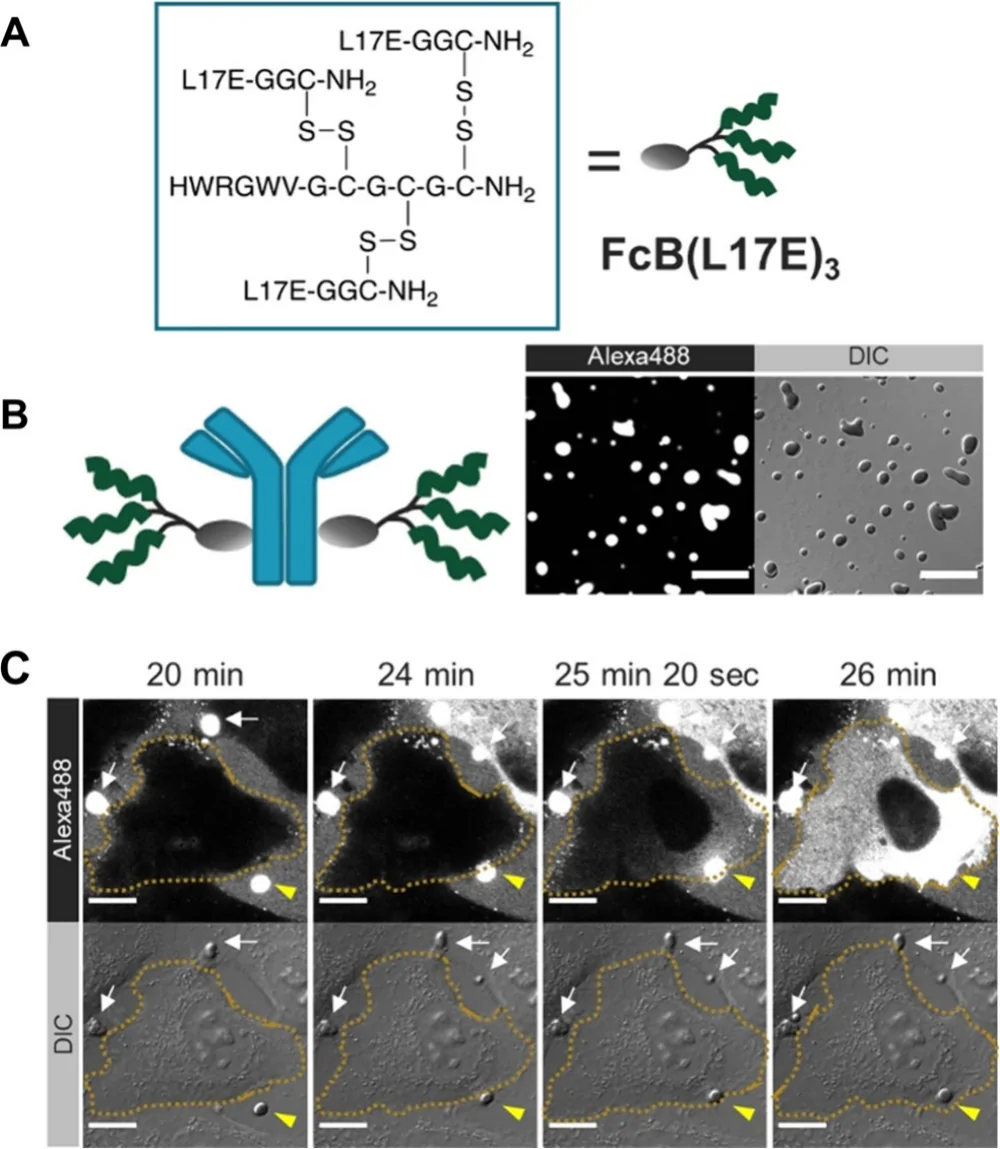
Trimerization of endosomolytic peptide L17E results in condensate formation with Alexa488-conjugated antibodies. (A). Structure of trimeric L17E. (B) Interaction of trimeric L17E with IgG. (C) Time-lapse imaging of IgG-L17E coacervate uptake. Adapted with permission from ref 166. Copyright 2021 John Wiley and Sons.

#### 3.2.2 Cellular Factors

Alongside the cargo and its delivery vehicle, the complex and dynamic nature of the cell is a critical parameter to consider when aiming for efficient protein delivery. This subsection specifically discusses the different cell-surface components that influence protein delivery strategies.

##### 3.2.2.1 Glycocalyx Layer

The most readily engaged cell-surface component for intracellular delivery is the cellular glycocalyx layer.^169–171^ This layer is composed of a dense forest of membrane-associated proteoglycans and free polysaccharide moieties on the extracellular side of the cell. Glycosaminoglycans (GAGs) found in the glycocalyx layer are composed of anionic sulfated disaccharides, such as chondroitin sulfate (CS) and heparan sulfate (HS), which act as a negatively charged shield directly over the cell membrane.^172^ For highly cationic delivery vectors, the glycocalyx serves as a critical initial docking site.^173–175^ Mislick and Baldeschwieler reported on the role of proteoglycans in the entry of gene complexes consisting of polycations and DNA as well as cationic lipids.^173^ Interestingly, this report states that the distribution of proteoglycans could explain the ease of transfection in certain tissue types. Subsequent work by Payne et al. extends this concept to cationic polymers and polypeptides.^175^ While these examples electrostatically interact with anionic polysaccharides, the guanidinium groups of arginine-rich peptides form bidentate hydrogen bonds with the sulfate, phosphate, and carboxylate moieties of the glycocalyx surface molecules.^176,177^ In addition to arginine interactions, Sagan and co-workers reported extensively on the role of hydrophobic tryptophan residues in strengthening the enthalpy of binding to the saccharide backbone via ion-pair π interactions (Figure 11).^178–180^ This binding mode leads to clustering of transmembrane proteoglycans, such as syndecans,^181^ thereby providing the necessary signal to trigger membrane invagination and active uptake.^182,183^ The molecular mechanism involved in proteoglycan-induced endocytic uptake has been extensively reviewed elsewhere.^175,182,184–187^

**Figure 11:**
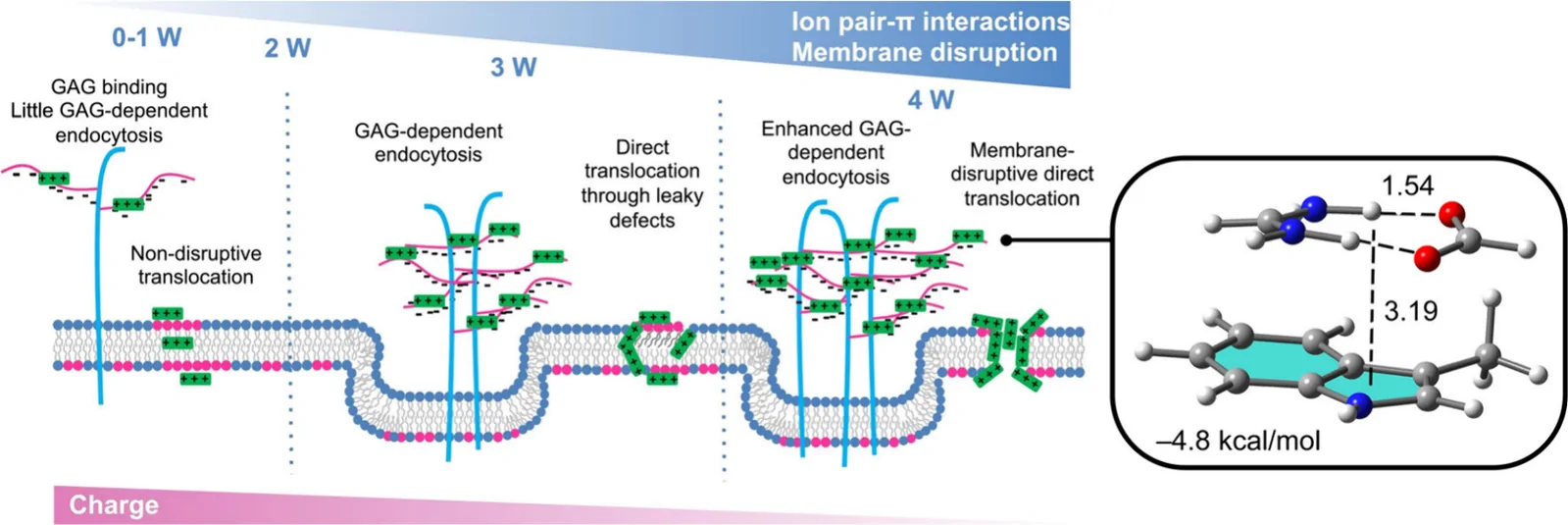
Model describing the internalization mechanism of Arg/Trp peptides. (Inset) Geometries of 3-methylindole with the formate anion-formamidinium salt-bridge. Adapted with permission from ref 179. Copyright 2020 Elsevier.

##### 3.2.2.2 Cell-Surface Proteins

Following the glycocalyx layer, cell-surface receptors are next encountered by cargo proteins. G-protein coupled receptors (GPCRs) are widely used as targets for intracellular delivery of proteins. Most examples that use GPCRs for the intracellular delivery of therapeutic agents are through antibody–drug conjugate (ADC) targeting.^188^ For protein delivery, photo-cross-linking approaches have been utilized to identify bona fide receptors for polyarginine CPPs, such as the chemokine receptor CXCR4 (Figure 12).^189,190^ CXCR4 was shown to stimulate R12 uptake by inducing macropinocytosis.^189^ Based on these findings in disease-related research, several studies have focused on targeting and activating CXCR4 to deliver protein cargos.^191–195^ Recent developments in photocatalytic proximity labeling technologies^196,197^ have provided further insights on potential cell–surface interactors of protein delivery vectors.^198^ In addition, by employing the biocompatible deazaflavin-diazirine energy-transfer labeling (DarT-labeling), intracellular interactors of cyclic versus linear CPP conjugates were identified, as well as the potential exocytosis of cyclic CPPs.^190^ These strategies aid in uncovering novel receptors and in understanding cellular uptake mechanisms to ultimately provide more efficient and targeted protein delivery.^190^

**Figure 12:**
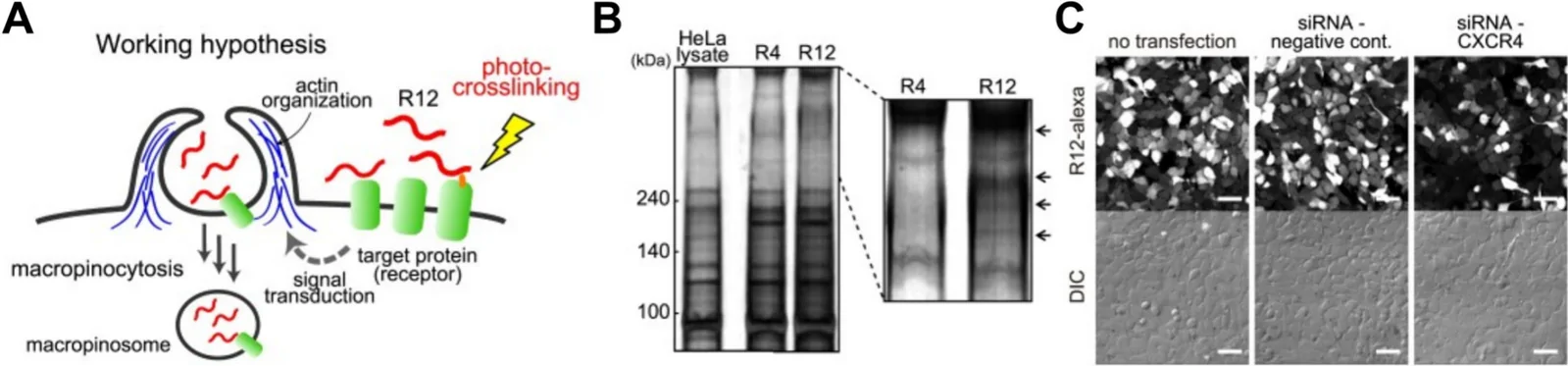
Photo-cross-linking revealed interaction between R12 and CXCR4. (A) Schematic diagram of photo-cross-linking of R12 on cell surface proteins. (B) SDS-PAGE analyses postphoto-cross-linking. Arrows indicate enriched proteins after R12 treatment. (C) R12 uptake in CXCR4-knockdown cells. Adapted with permission from ref 189. Copyright 2012 Elsevier.

Similarly, researchers have decorated delivery vehicles with receptor-binding compounds or biomacromolecules that activate receptor tyrosine kinases and integrins.^199–201^ Lieser et al. reported the combination of an epidermal growth factor receptor (EGFR)-targeting moiety and endosomolytic peptides for targeted delivery in breast cancer cells.^168^ Mechanistically, EGFR engagement activates receptor-mediated endocytic uptake together with the intended protein target, in this case, mCherry. Upon endosomal acidification, the endosomolytic peptides facilitated the release of the protein cargo into the cytoplasm. EGFR targeting has also been reported for the delivery of extracellular vesicles,^202^ lipid nanoparticles,^203^ and polymers.^204^ Integrins, on the other hand, are generally targeted using the Arg-Gly-Asp (RGD) tripeptide.^205^ This tripeptide has been extensively used for the delivery of a multitude of cargoes ranging from small molecules to proteins,^206–209^ as well as in combination with other intracellular delivery molecules that improve uptake.^210–213^

Cell-surface proteins, including integrins, protein disulfide isomerase, and transferrin receptor, play a pivotal role in thiol-mediated uptake, acting as selective thiol/disulfide exchange partners and forming covalent networks that drive substrate translocation across the membrane (see section 4.3).^214,215^

##### 3.2.2.3 Cell Membrane Lipid Composition and Dynamics

Below the glycocalyx layer, we find the cell membrane, which drastically affects protein delivery into cells, as previously stated.^216–218^ Structurally, it is characterized as a dynamic fluid mosaic of lipids and proteins that forms a hydrophobic film, which inherently restricts the passive diffusion of large, polar macromolecules, including most therapeutic proteins, due to their large hydrodynamic radius.^25,170,219,220^ For this review, we will exclusively focus on cell membrane lipids composition and dynamics relevant for protein delivery.^218,221,222^

Early cell penetration studies demonstrated the influence of lipid composition on intracellular uptake.^223,224^ Although initial work focused largely on penetratin translocation across a pure lipid bilayer with a uniform composition, later reports combined a variety of lipid compositions to assess CPP uptake. A specific example is a study by Pooga and co-workers, in which they reported the entry of arginine-rich CPPs into lipid vesicles with lower cholesterol content.^225^ This concept was modeled by Akiyama and co-workers, who reported a suitable membrane model that can be used to predict the translocation process.^226^ Aside from cholesterol content, Brock and co-workers reported the influence of sphingomyelin-to-ceramide conversion on polyarginine uptake.^227,228^ The group considered that the CPP-induced translocation of acid sphingomyelinases to the outer leaflet of the cell membrane, and eventual conversion of sphingomyelin to ceramide, are determinants of CPP translocation and uptake into the cell.^227^ Later, the group also highlighted that this enzymatic conversion to ceramide is the key driver of translocation.^228^ Along these lines, Choi and co-workers used giant unilamellar vesicles (GUVs) and reported that nonaarginines (R9) form electrostatic interactions with anionic phosphatidylserine (PS)-rich domains on the outer leaflet of the cell membrane. This interaction fluidizes the PS-rich domains, forming liquid-disordered domains and leading to peptide permeabilization.^229^ In parallel, Povilaitis and Webb also showed that cationic membrane-penetrating peptides (NAF-1 ^44–67^ and R_6_W_3_) exerted enhanced insertion into model membranes containing anionic lipids, phosphatidic acid (PA), phosphatidylglycerol (PG), and PS.^230^ Although these investigations showed how anionic lipid composition in model membranes may contribute to protein delivery using cationic vectors, the outer leaflet of mammalian cell membranes is largely composed of neutral phospholipids, with only around 2–3% of anionic lipid. Thus, the overall contribution of anionic lipids in a cellular setting should be critically considered.^231^ All these studies suggest that membrane composition influences membrane dynamics, specifically membrane fluidity, which we will further discuss below. We refer readers to other dedicated reviews for more thorough discussions of the complex relationship between the lipid landscape and protein uptake.^232–234^

Given that the cell membrane is the primary barrier to the intracellular delivery of exogenous materials, its architecture and dynamics should also play a significant role. Through biophysical assessments, several studies point toward the ability of cationic CPPs to induce membrane curvature.^235,236^ Later, membrane curvature was found to be involved in protein delivery.^103,237^ Co-addition of the membrane curvature-sensing peptide EpN18 led to the formation of positive membrane curvature and more efficient uptake of octaarginine (R8) CPP by direct translocation^238^ and by endocytic uptake.^239^ This concept was adopted by other groups.^240–242^ Octaarginines (R8) were found to translocate through areas of the membrane with loose lipid packing (Figure 13).^243^ It is believed that the loosening of membrane lipid packing enhances the interaction between the R8 backbone and the lipid acyl chains, resulting in peptide translocation.^243^ A recent study also showed that other CPPs, namely penetratin, insert themselves between lipid molecules in a concentration-dependent manner and influence lipid packing density.^244^ Although these primary studies focused exclusively on peptide translocation, enhanced delivery of GFP-R8 in the presence of pyrenebutyrate (PyB) suggests the influence of this lipid membrane dynamic on protein delivery via direct translocation.^245^ Lastly, membrane composition could also change membrane fluidity. This is also evident in reported cases in which lipid mixing via “flipping” alters the outer leaflet landscape sufficiently to influence the uptake of CPPs.^246,247^ These works emphasize the importance of the membrane state (composition and architecture) in studying the uptake of the cargo proteins.

**Figure 13:**
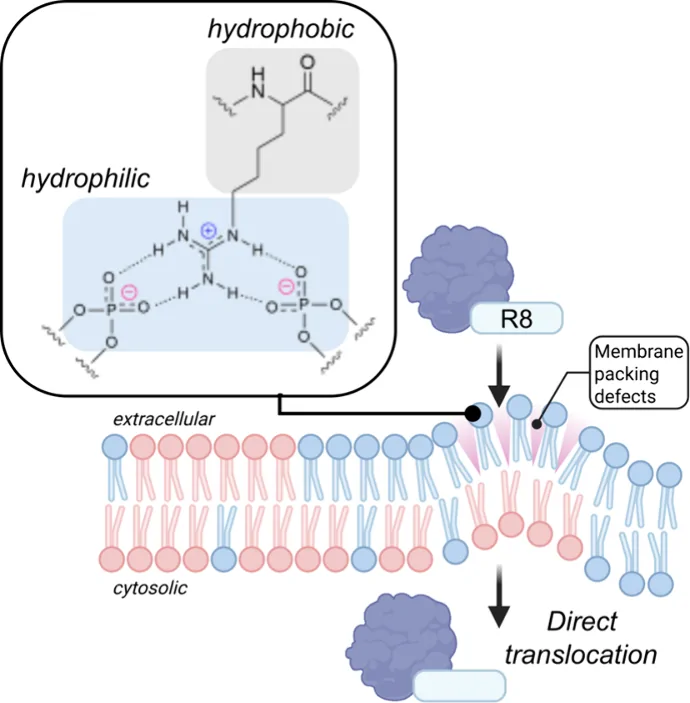
Membrane interaction and lipid packing defect affect protein uptake into cells. (Inset) Interaction of guanidinium groups from arginine and phosphate groups from cell membrane lipids. Highlighted are distinct hydrophobic and hydrophilic areas. Membrane packing defects are believed to be sites for direct translocation of R8 CPP and CPP-conjugated proteins. Created with https://BioRender.com.

##### 3.2.2.4 Membrane Potential

The difference in electrical potential between the cellular interior and exterior, namely, the membrane potential, was proposed as an important factor shortly after the discovery of cationic CPPs. In 2003, Lindblom et al.^248^ and Terrone et al.^224^ reported membrane-potential-dependent uptake of polyarginine peptides in large unilamellar lipid vesicles. This was complemented by Wender and co-workers in 2005, who demonstrated the decisive role of the cell membrane potential in the direct translocation of guanidinium-rich peptides into Jurkat cells (Figure 14).^176^ Several groups then independently validated the decisive role of membrane potential for the peptide uptake using various delivery vectors.^249,250^ It has been proposed and computationally modeled that this is tightly linked to the formation of water pores through the membrane of cells, allowing for the translocation of cargo.^134,251^ Only recently, it was demonstrated that even protein cargoes can directly translocate through membrane-potential-driven water pores.^137^ Water pore formation is shown to be a consequence of changes in the local transmembrane electric field induced by the accumulation of positively charged peptides on the negatively charged membrane (Figure 14). This topic will be further discussed in section 4.3.

**Figure 14:**
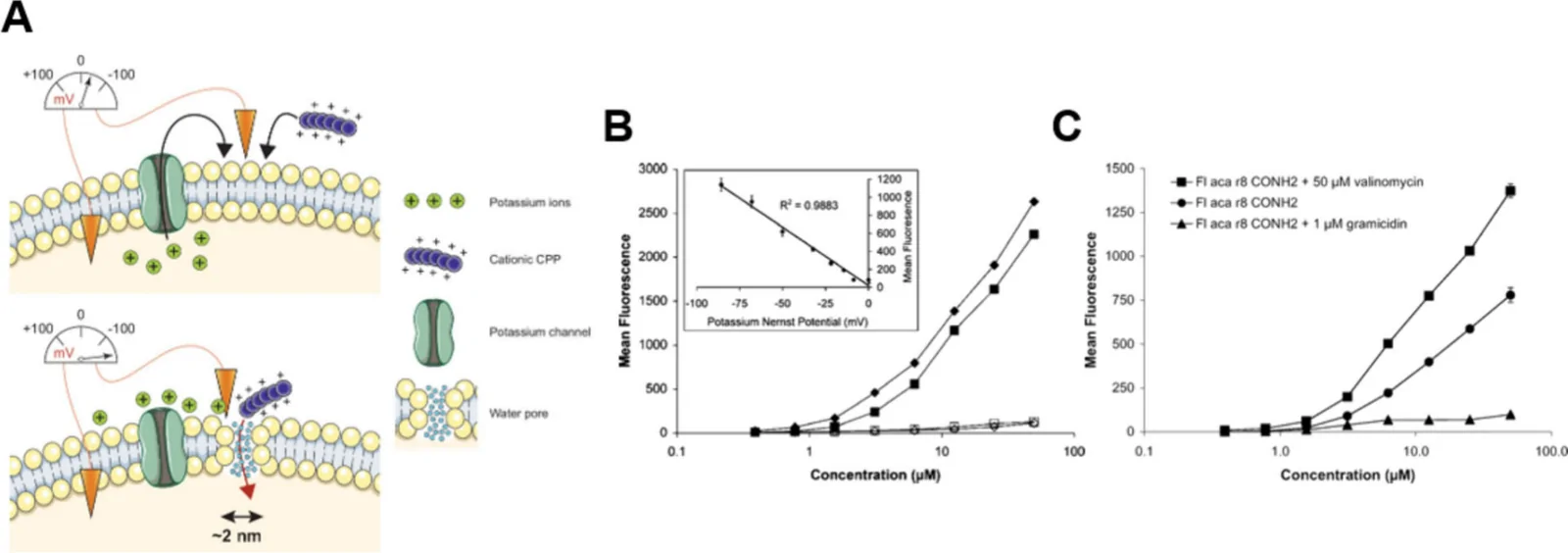
Membrane potential influences direct translocation of peptides. (A) Cationic CPP translocation across cellular membranes is favored by megapolarization. A low membrane potential is permissive for direct CPP translocation. Cationic CPPs binding to polarized membranes induce further decrease of local potential, which leads to the formation of water pores that are then used by CPPs to enter cells. Reproduced with permission from ref 134. Copyright 2021 The Author(s), Creative Commons CC-BY license. (B,C) Graphical representation of increasing and decreasing resting membrane potential to overall direct translocation of model peptides. Reproduced from ref 176. Copyright 2004 American Chemical Society.

#### 3.2.3 Environmental Factors

Lastly, the extracellular microenvironment in which the cargo proteins and vectors exist also plays a crucial role in the efficiency and effectiveness of protein delivery.

Although temperatures are tightly regulated and mostly stable *in vivo*, benchtop cellular assays experience considerable temperature fluctuations that can affect experimental outcomes. The temperature can easily be manipulated for *in cellulo* experiments resulting in decreased energy-dependent uptake, hence altering overall uptake. Therefore, experiments at 4 °C have widely been used to study and validate direct translocation pathways.^252,253^ However, low temperatures also increase membrane rigidity, suppress metabolic fluxes, slow enzymatic kinetics and ion transport, and reduce diffusion rates, thereby impairing all delivery processes.^100,254–256^ Hence, results from temperature manipulation experiments to study cellular uptake warrant careful interpretation.

The extracellular pH modulates the protonation state of the cargo protein, the delivery vector, and the cellular matrix (phospholipids, sugars), thereby altering their interactions. In particular, for charged delivery vectors, extracellular pH critically governs efficiency by modulating protonation states that drive vector-cell interactions and cellular entry.^257^ Furthermore, the pH also alters the nucleophilicity of cell-surface nucleophiles most prominently cysteine residues, affecting thiol-dependent uptake mechanisms.^34,214^ An important and often addressed scenario is the acidic tumor microenvironments,^258^ which was used for targeted delivery. Lower pH values can, for example, be used to activate delivery vectors in specific cellular environments, as demonstrated by activatable cell-penetrating peptides.^259^

Besides the crowded extracellular matrix, the composition of the extracellular fluid, in particular serum proteins, strongly affects cellular delivery. While interactions with serum proteins can increase the circulation time of smaller delivery vectors,^260^ they can also inhibit their capacity for protein delivery into target cells due to nonspecific noncovalent or covalent interactions.^261^

### 3.3 Analysis and Characterization of Protein Delivery Strategies

As discussed above, the internalization of proteins can follow multiple uptake pathways and is governed by various parameters. While there have been significant advances in the field to improve cellular delivery of functional proteins, accurate and efficient characterization of the delivery process is not straightforward. One general challenge arises from the need to differentiate between cytosolically available protein and cargo that remains sequestered within endolysosomal compartments or is nonspecifically associated with the cell surface. Another factor that must be considered is the consequence of introducing tags necessary to monitor protein delivery, e.g., fluorophores or protein fragments, which might alter the properties of the cargo (surface charge, hydrophobicity, size, etc.) as well as its interactions with the membrane and potential targets. Furthermore, unlike small molecules and peptides, the tertiary structure of proteins must be preserved to ensure their functionality upon delivery. And of course, it should also be validated that the observed delivery does not result from experimental artifacts such as compromised membrane and reduced cell viability. This section aims to provide an overview of common analytical assays used to evaluate the delivery of modified proteins while highlighting some technical challenges and potential pitfalls associated with these methods.

#### 3.3.1 Analyzing Uptake Pathways

One fundamental step in characterizing any protein delivery system is determining whether the cargo enters the cell via an active vesicle-mediated endocytic pathway or by direct translocation across the plasma membrane. While direct translocation is highly desirable for immediate cytosolic bioavailability, most protein–carrier conjugates are internalized through endocytic pathways. To differentiate these mechanisms, researchers have employed a suite of assays, many of which were originally developed for studies of CPP uptake and have been extensively reviewed elsewhere.^262,263^ However, because the protein cargo itself significantly influences the physicochemical properties of the delivery system, results obtained from isolated CPP studies cannot be directly extrapolated to CPP-mediated protein delivery.^153^ Increased size, altered surface charge, and greater structural complexity of protein cargo can change the internalization route and overall delivery efficiency. Table 1 summarizes the most common characterization techniques of uptake pathways within the context of the protein delivery platforms covered in this Review. It is important to note that, while various experimental approaches are available for investigating cellular uptake mechanisms, no single method is neither universally applicable nor sufficient. The choice of strategy must be tailored to the specific research question, and previously established protocols should be critically evaluated for their suitability and limitations in a given experimental context. Importantly, fixation of cells prior to analysis has generated misleading results in the past, especially concerning free CPPs, as they are now known to redistribute between cellular compartments even upon mild fixation.^252,264^ While it was reported that no such effect was observed for CPP–cargo conjugates, it is necessary to always validate successful delivery in living cells in order to exclude potential fixation artifacts.^265^

**Table 1:** Common Methods for Identifying the Predominant Mechanism of Protein Delivery

assay type	principle	caveats	protein cargo
**Uptake Inhibition**	Low Temperatures	Affects cellular physiology, membrane biophysics and peptide-membrane interactions	GFP^277–282^
			Nanobody^29,34^
			Antibody^34^
			mCherry^34,167^
			BSA^283^

	ATP-Depletion	Cellular stress	Cre^153^
			Antibody^35^
			BSA^283^

	Chemical Inhibitors of Endocytosis	Side-effects and varying efficacy between cell lines	Cre^153,284^
			GFP^282,285^
			BSA^283^

	Genetic Inhibition of Endocytic Machinery	Multiple processes might be affected	Cre^181^

**Colocalization**	Endosomal Markers	Any punctate signal that does not colocalize with common markers can be mischaracterized as endosomal escape	Saporin^286^
			Avidin, Streptavidin^287^
			Cre^181^
			BSA^283^

	Endosomal Rupture	Does not account for transient endosomal leakage	mCherry^157^

**Uptake Kinetics**	Calcein Coincubation	Lack of standardized protocol; high concentration required for self-quenching	GFP^275^
	Activatable Fluorophores	Hydrophobic dyes might affect permeation properties	Nanobody^137^

To leverage the most fundamental distinction between endosomal and direct translocation pathways—the former being an energy-dependent process involving membrane remodeling into vesicles, while the latter is largely energy-independent—a common approach is to deplete the system of required energy.^100^ This is typically achieved by performing delivery experiments at low temperatures (4 °C) or by adding reagents such as sodium azide or 2-deoxy-d-glucose to deplete the ATP required for active membrane rearrangement in energy-dependent processes (Figure 15). Comparing delivery efficiency under these inhibitory conditions with physiological controls provides the first indication of whether the uptake mechanism is predominantly endosomal or involves some form of direct translocation. However, this approach can yield ambiguous results and is prone to overinterpretation. As mentioned above, low temperatures not only affect ATP-dependent endocytic processes but also other aspects of cellular physiology as well as membrane biophysics and peptide/protein–membrane interactions.^256^ It also has a diminishing effect on direct translocation, so temperature-dependent experiments should ideally be interpreted only with complementary mechanistic assays.^100^ Small-molecule inhibitors of endocytosis are widely used tools for probing specific uptake pathways.^266^ However, their efficacy and cytotoxicity are highly cell type dependent, and many show off-target effects that might undermine or produce conflicting results.^267,268^ Thus, procedures optimized in one cell line cannot be directly transferred to other cell lines without appropriate controls. For more thorough mechanistic studies, alternative approaches such as genetic knockout or knockdown of key internalization components or overexpression of dominant-negative mutants involved in the process can provide more specific evidence.^134,181,266,269,270^

**Figure 15:**
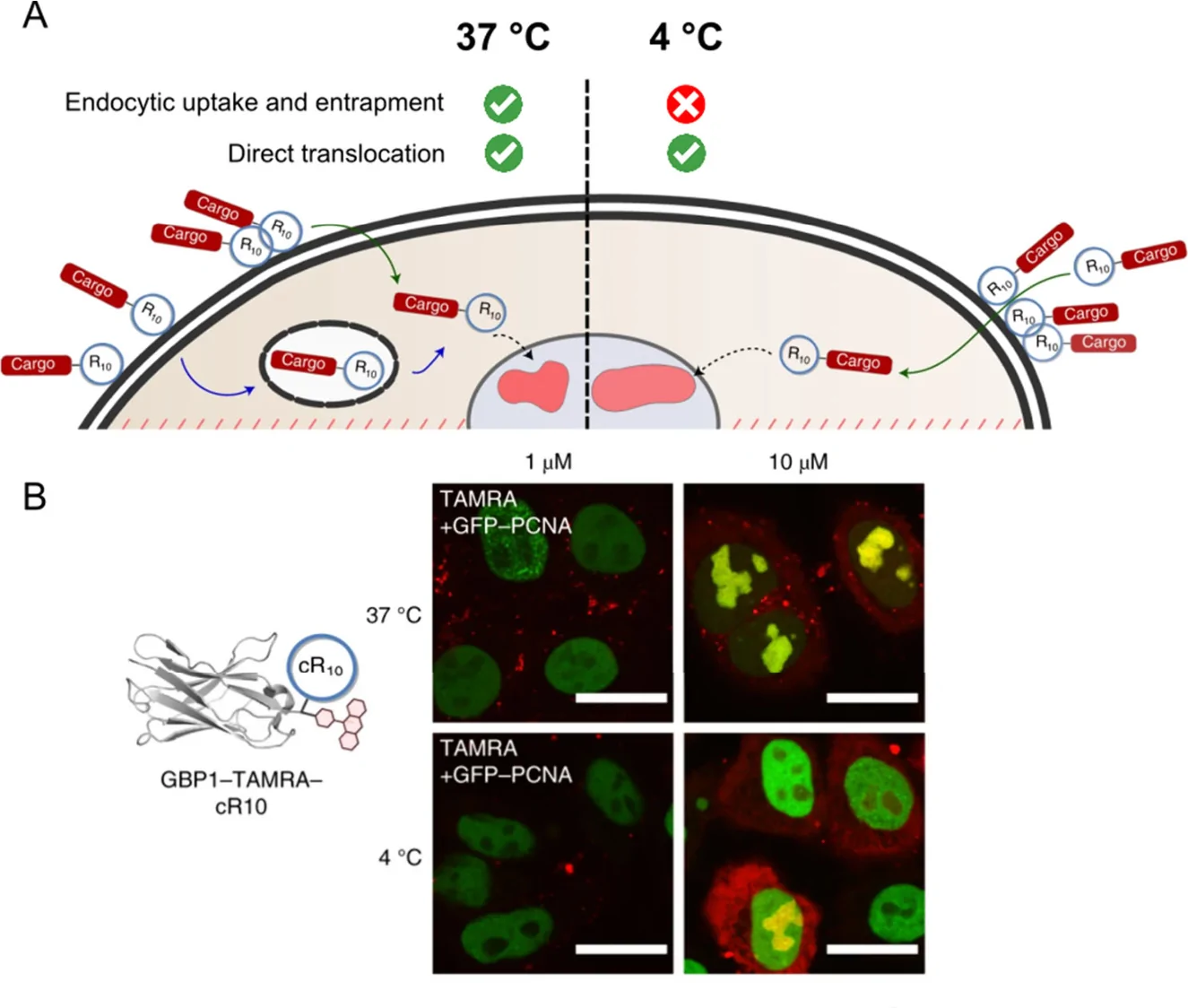
Effect of temperature on protein delivery. (A) Schematic diagram of temperature-dependent intracellular entry pathways. (B) representative image of GBP1-TAMRA-cR10 uptake at 37 °C showing more punctuate endosomal signals than at 4 °C. Adapted with permission from ref 34. Copyright 2021 Springer Nature.

While the aforementioned inhibition studies can be performed with various analytical readouts, fluorescence microscopy offers unique advantages for probing pathway mechanisms that are difficult to achieve with other techniques. For instance, mechanistic insights are frequently derived from colocalization assays utilizing commercially available live-cell probes (e.g., LysoTracker and pHrodo), established markers of specific endocytic routes (e.g., transferrin for clathrin-mediated endocytosis, dextran for macropinocytosis, and cholera toxin B for clathrin-independent pathways), or distinct protein markers such as Rab5/EEA1 (early endosomes), Rab7 (late endosomes), and Lamp1 (lysosomes) (Figure 16).^266,271,272^

**Figure 16:**
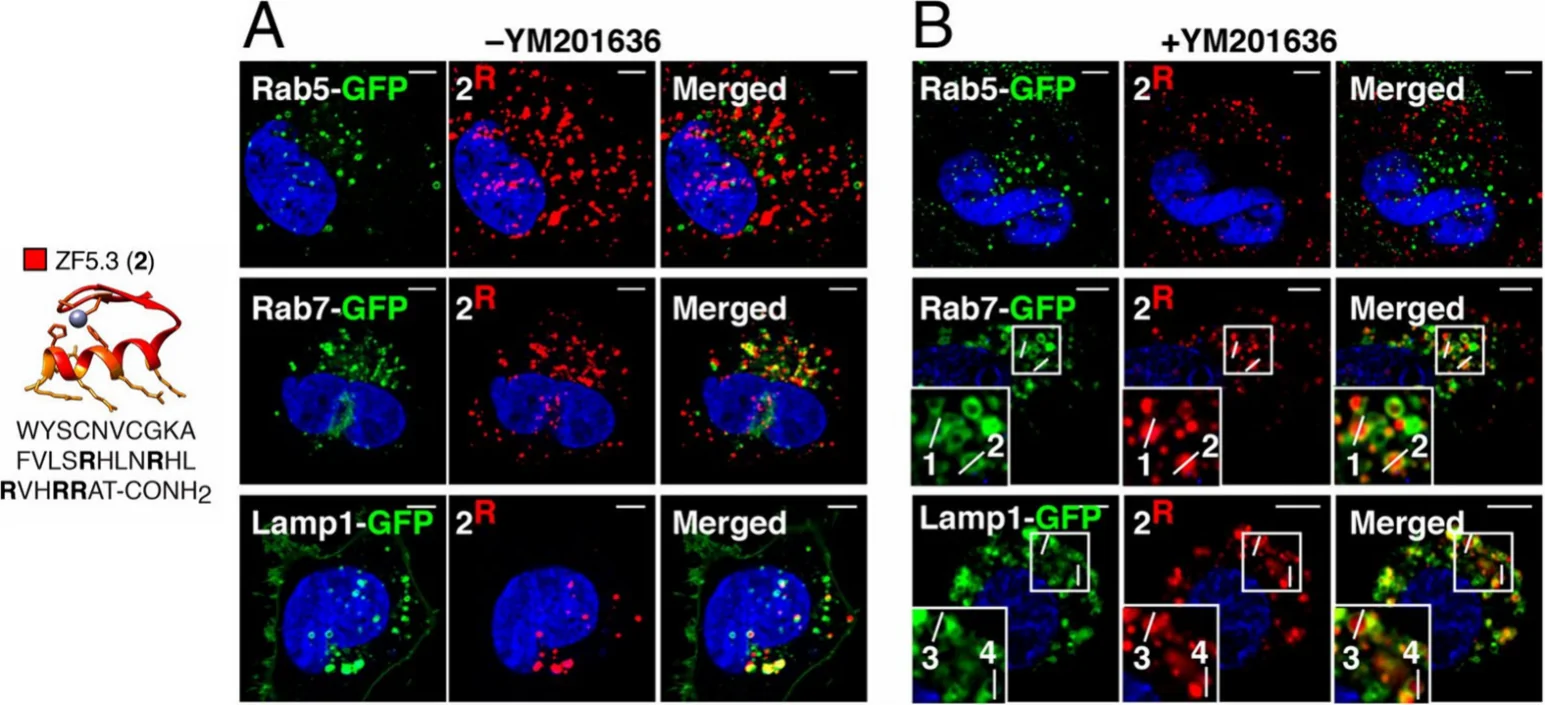
Colocalization of ZF5.3 with classic endocytosis markers Rab5, Rab7, and Lamp1. Confocal images of uptake (A) in the absence or (B) in the presence of an inhibitor. Adapted with permission from ref 271. Copyright 2019 National Academy of Sciences.

Alternatively, reporter cell lines expressing GFP-tagged Galectin-3 (Gal3) can be used to specifically evaluate whether endosomal rupture takes place at any time point during delivery.^272^ Upon cellular expression, Gal3-GFP is distributed throughout the cytosol; however, once the endosomal rupture occurs, it is recruited to exposed luminal β-galactosides, transforming from a diffuse signal into a distinct punctate fluorescent pattern (Figure 17).^112,157,273^ Furthermore, fluorescence readout can provide time-resolved information on cargo entry and its transition between different compartments. Since endosomal uptake and escape occur over a longer timescale (>5 min) than near-instantaneous direct translocation, these kinetic differences can be used to distinguish the two pathways.^22^

**Figure 17:**
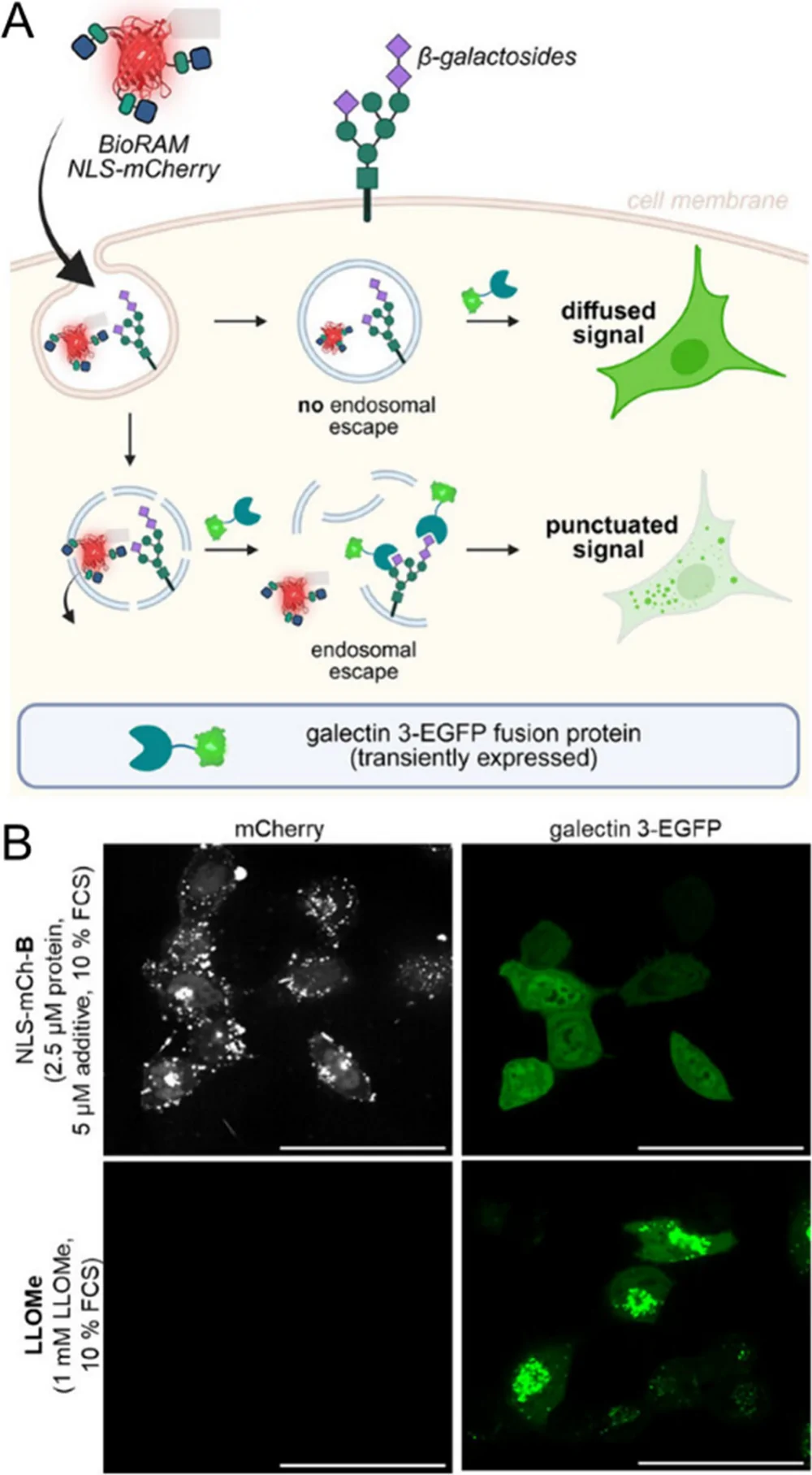
Gal3-GFP assay to evaluate endosomal escape. (A) Mechanism of Gal3-GFP accumulation assay. (B) Representative data showing the delivery of BioRAM-conjugated mCherry (NLS-mCh-**B**) without observing endosomal rupture. The lower panel shows positive control LLOMe-treated cells resulting in endosome rupture. Adapted with permission from ref 157. Copyright 2025 The Author(s), Creative Commons CC-BY license.

Still, differentiating between endosomal and cytosolic signals can be challenging and prone to artifacts, even with confocal setups. Therefore, the use of cytosolically activatable signals, which remain less or nonfluorescent until they reach the environment of cytoplasm, can provide the advantage of a defined, endosome-free signal. Historically, such approach has been applied in calcein leakage or codelivery assay, which exploits concentration-dependent self-quenching of the dye. The result is a weak fluorescent signal at high dye concentrations such as found in endosomes, and a bright fluorescent signal once the dye reaches cytosol.^274^ At concentrations below the self-quenching threshold, calcein can be helpful to track both endosomal and cytosolic signals, which has also been used to analyze CPP-based protein delivery systems and their kinetics.^275^ Another approach developed by the Langel lab involves a disulfide-linked fluorophore-quencher pair attached to a CPP: upon entry into the cytoplasm, the disulfide bond is cleaved, releasing the quencher, which results in a fluorescent signal allowing for quantification of uptake in real time.^276^ A similar approach has been recently reported for activatable protein cargo employing brighter fluorophores with enhanced turn-on and decreased background, enabling confocal time-lapse microscopy to visualize immediate direct translocation at the cellular level.^137^ While these strategies have been utilized for mechanistic characterization, many other cytosol-sensitive tags have been developed with the primary goal of quantifying the amount of delivered cargo, which will be covered in the following section.

#### 3.3.2 Quantifying Delivery Efficiency

An accurate assessment of cytosolic delivery is essential for the rational design and optimization of protein delivery platforms. Unlike small-molecule compounds, which often rely on passive diffusion that can be modeled using artificial membranes or liposomes, macromolecules such as peptides and proteins require a live-cell environment for accurate evaluation since several parameters influencing protein uptake are not adequately captured by simplified model systems. While the analytical toolbox for quantification of small molecule and peptide uptake is diverse and has been reviewed extensively elsewhere,^288^ only a fraction of these methods have been applied to protein delivery. Table 2 highlights the techniques discussed in this section, categorized according to their level of quantification.

**Table 2:** Common Methods for Quantification of Protein Delivery Using Methods Covered in This Review

quantification level	assay method	primary readout	key aspects	protein cargo
**Total Uptake (semiquantitative)**	Automated Microscopy and Image Analysis	Fluorescence	- Prone to artifacts; requires validated segmentation scripts; (activatable) fluorescent tag can influence uptake, cargo trafficking or folding	mCherry^34,137,157,167^
			+ Easy to set up	

	Flow Cytometry	Fluorescence	- No differentiation between compartments; endosomal uptake inhibitors or activatable fluorescent tag have to be included to measure cytosolic signal with their own caveats	SNAPtag, APEX2 ^291^

			+ Easy to set up	HaloTag^295^
				Effector proteins^293^
				mNeonGreen^309^

**Cytosolic Uptake**	Fluorescence Correlation Spectroscopy (FCS)	Fluorescence	- Technically challenging; low-throughput; fluorescent tag required can influence uptake, cargo trafficking or folding	SNAPtag, APEX2 ^291^
			+ Absolute quantification possible	Argininosuccinate Synthetase^290^
				DHFR enzyme^292^

	Split GFP	Fluorescence	- Low sensitivity (>0.1 uM range); 16 amino acids-tag can influence uptake, cargo trafficking or folding; stable cell line required	TRX^297^
			+ Absolute quantification possible	Antibody^298^

	Split NanoLuciferase (SLEEQ)	Luminescence	- 11 amino acids-tag can influence uptake, cargo trafficking or folding; stable cell line required	GFP^141^

			+ pM sensitivity; absolute quantification possible; quantification of endosomal escape efficiency	Antibody^299,300^
				Cre^301^

	Glucocorticoid Receptor Translocation	Fluorescence	– Lipophilic tag that can influence uptake; relative, indirect readout	scGFP^142^
			+ Small-molecule tag	

	Chloroalkane HaloTag Azide-Based Membrane Penetration (CHAMP)	Fluorescence	- Requires unnatural amino acid incorporation or protein–surface modification with azide tag; indirect, readout; stable cell line required	GFP^303^
			+ Very minimal tag	

	Biotin Ligase (BirA)	Western Blot	- Low throughput; 15-amino-acid tag; stable cell line required	DARPin^305,306^
			+ Absolute quantification possible; quantification of endosomal escape efficiency	

	CytoSNAP	Luminescence	- Low throughput; stable cell line required	STxB^307^
			+ Absolute quantification possible; quantification of endosomal escape efficiency	

For any delivered molecule, accurate subcellular localization is essential since activity is generally compartment-specific. In particular, proteins trapped on the outer cell membrane or within endosomes can confound readouts and lead to overestimation of performance. While fluorescence microscopy is a common and accessible approach, conventional setups usually record total internalized cargo and lack the high-throughput capabilities of methods such as flow cytometry. These limitations can be partially circumvented by employing confocal microscopy setup, automated acquisition and intensive image processing. By excluding endosomal puncta and averaging over hundreds of individual cells, this approach can allow for a statistically significant. readout.^157,167,289^ Nevertheless, it remains prone to artifacts, such as background or out-of-focus endosomal signals and imprecise segmentation, and is therefore considered semiquantitative. A more rigorous alternative to fluorescence microscopy is Fluorescence Correlation Spectroscopy (FCS), which can determine cytosolic concentration from the unique diffusion signature of fluorescent cargo (Figure 18). By analyzing fluorescence intensity fluctuations within a microscopic focal volume, FCS determines local concentration independently of sequestered endosomal signals. While this provides a highly specific measure of cytosolic protein cargo, the method is technically demanding and low-throughput.^290–292^ To provide a complete profile, it is therefore often used in tandem with flow cytometry to capture the total cellular uptake.

**Figure 18:**
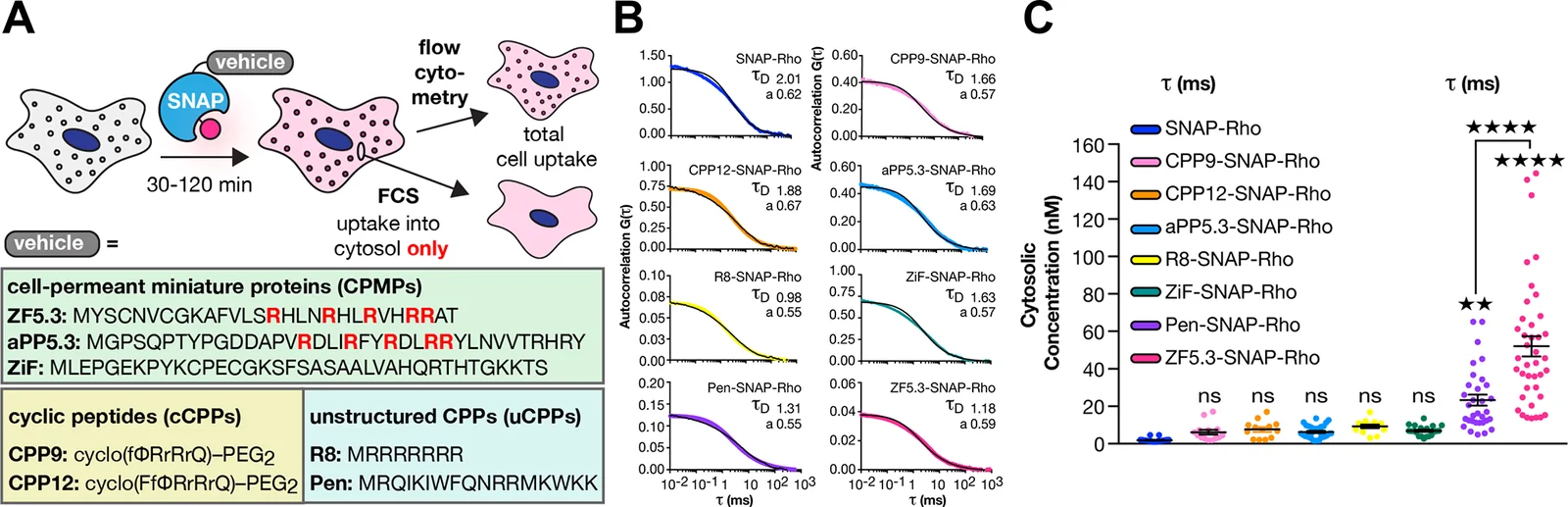
Schematic representation of FCS-based quantification of cytosolic delivery of SNAP-CPP conjugate. (A) Mechanistic diagram and list of analyzed vehicle peptides. (B) Raw FCS data used to calculate (C) the concentration of cytosolically available SNAP-vehicle conjugate. Reproduced from ref 291. Copyright 2018 American Chemical Society.

Flow cytometry, on the other hand, is the standard method for analyzing thousands of cells in a high-throughput manner; however, conventional setups do not distinguish between membrane-bound, endosomal, or cytosolic internalized signals. Extracellular quenching and trypsinization, combined with small-molecule inhibitors of endocytosis, can eliminate surface-bound and endosomal signals, respectively, ensuring that only fluorescence originating from direct translocation is recorded.^293^ Alternatively, for both confocal microscopy and flow cytometry, cytosol-sensitive fluorescent tags provide a major advantage. In addition to the fluorophore–quencher pairs, pH-sensitive systems are effective tools for isolating the cytosolic signal. For example, Pei and co-workers utilized a pH-sensitive naphthofluorescein dye conjugated to CPPs that remains nearly nonfluorescent under the acidic conditions of the endolysosomal compartment (pH < 6.0) but becomes highly fluorescent upon reaching the neutral cytosol (pH 7.4).^294^ Similar approaches have recently been adapted for the quantification of protein uptake.^293,295^ Also, fluorogenic probes that are activated by cytosolically expressed enzymes have been explored for protein delivery quantification.^296^

While environment-sensitive fluorescent probes provide a rapid response signal, background from endosomal compartments or fluorophore leakage upon partial degradation cannot be completely excluded. Split-protein complementation assays address these limitations by combining an activatable approach with biological gate-logic that requires spatial proximity for signal generation. In this approach, a fluorescent or luminescent protein is split into two nonfunctional fragments, with the larger fragment typically expressed stably in the cytosol of a reporter cell line while the smaller tag is conjugated to the cargo. Signal is generated only upon successful delivery to the cytosol, where the two fragments spontaneously reassemble to reconstitute the active full-length protein. The first such system applied to protein delivery quantification utilized split-GFP fragments and flow cytometry as a readout, while the principle also allows for use with a microplate reader (Figure 19A).^297^ Subsequent developments focused on enhancing the sensitivity of this method to >0.1 μM by including streptavidin-based interactions. Generation of a GFP standard curve and complementary quantitative Western blotting allowed for total uptake and endosomal escape quantification in absolute concentration values.^298^ To further increase sensitivity, similar gate logic was applied using a split nanoluciferase (NanoLuc) enzyme, which generates a bright luminescence signal upon the addition of a cell-permeable substrate. This system enabled evaluation of endosomal escape for model proteins at picomolar concentrations, although only in terms of relative uptake amounts (Figure 19B).^141,299,300^ Recently, this approach was extended to allow for absolute concentration determination of cytosolic protein delivery through the use of a luminescence calibration curve, providing a highly sensitive alternative to traditional fluorescence-based assays.^301^

**Figure 19:**
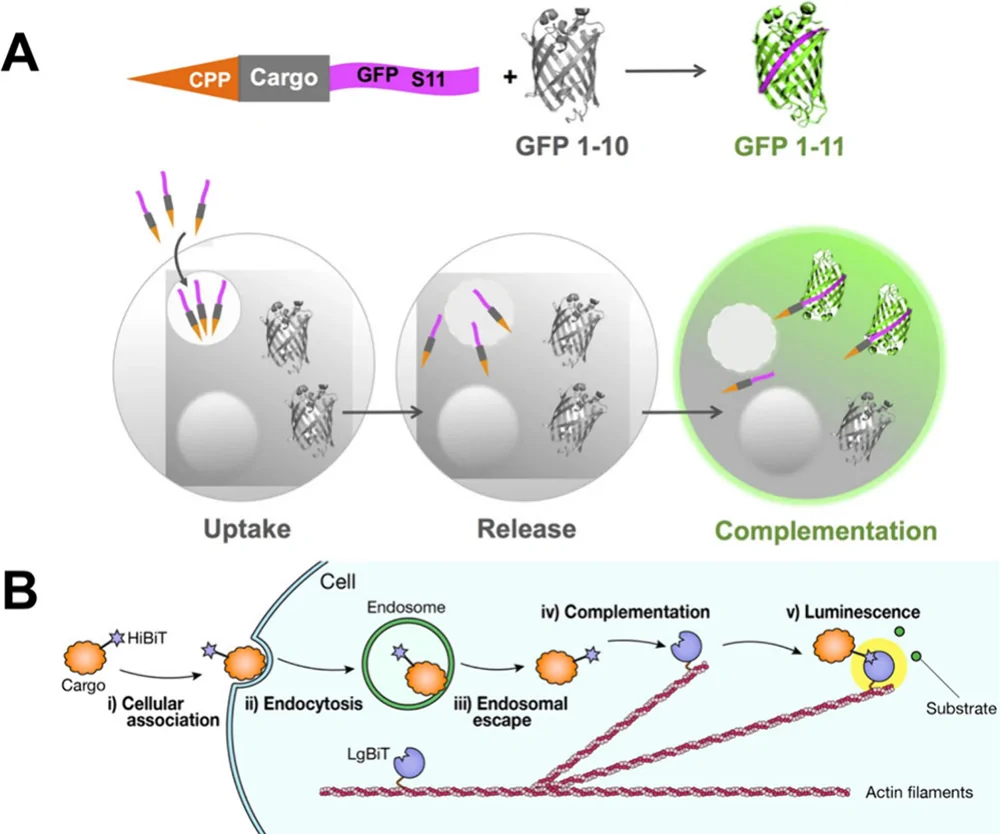
Schematic representation of (A) split GFP-based (Reproduced with permission from ref 297. Copyright 2015 The Author(s), Creative Commons CC-BY license), and (B) split luciferase-based quantification of cellular delivery (Reproduced with permission from ref 141. Copyright 2021 The Author(s), Creative Commons CC-BY license). Uptake efficiency is indirectly measured by the recovery of fluorescence or luminescence, respectively.

Other reporter systems include cells transfected to express the cytosolic glucocorticoid receptor (GR) fused with a fluorescent protein for microscopy readout. A protein of interest is modified with a small-molecule tag dexamethasone, which, upon successful delivery, activates GR and results in its nuclear-to-cytoplasmic translocation that can be quantified as a ratio.^142,302^ However, dexamethasone being a cholesterol derivative is therefore quite lipophilic, which can have an influence on the delivery process. An alternative approach is provided by the Chloroalkane HaloTag Azide-Based Membrane Penetration (CHAMP) assay, an extension of the Chloroalkane Penetration Assay (CAPA) method, widely used for small-molecule quantification.^303^ CAPA relies on a pulse-chase mechanism where a chloroalkane-tagged cargo competes with a fluorescent tracer for binding sites on a cytosolically expressed HaloTag (Figure 20A).^304^ CHAMP adapts this for proteins using a minimal azide tag, incorporated via unnatural amino acids or NHS-azide modification (Figure 20B). Similar to the GR assay, the CHAMP approach currently provides only a relative and indirect quantification of cytosolic delivery (Figure 20C).

**Figure 20:**
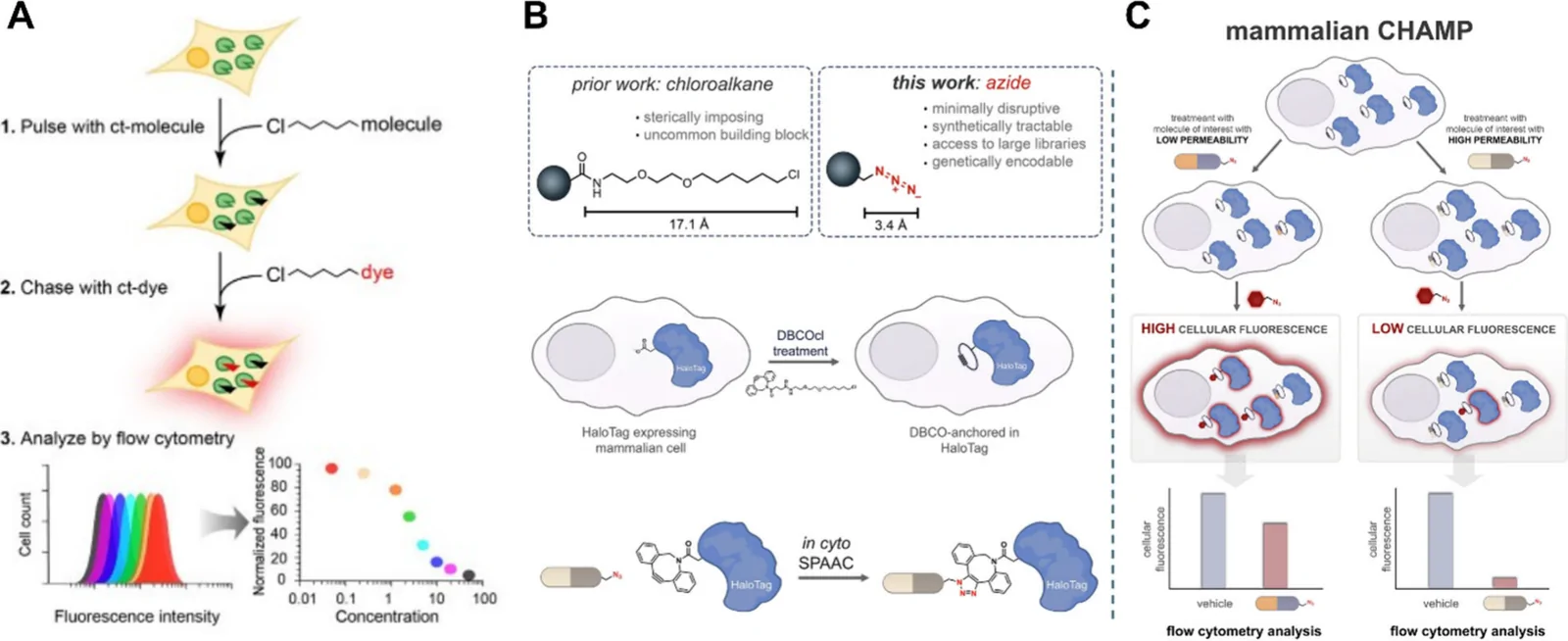
Chloroalkane Penetration Assay (CAPA) and Chloroalkane HaloTag Azide-based Membrane Penetration (CHAMP) assay. (A) Schematic diagram showing the CAPA process and data analysis. Reproduced from ref 304. Copyright 2018 American Chemical Society. (B) Structural size comparison between chloroalkane (HaloTag) and azide quantification tags. Installation of DBCO-chloroalkane into HaloTag allows cytosolically delivered N_3_-modified proteins to react with the strained alkyne handle via SPAAC (pulse) and subsequent incubation with N_3_-dye (chase) reports on cytosolic localization via flow cytometry. (C) Schematic representation of mammalian CHAMP workflow. Reproduced with permission from ref 303. Copyright 2021 The Author(s), Creative Commons CC-BY license.

Assays that offer absolute quantification of both cytosolic and total delivered cargo include the biotin ligase (BirA) assay (Figure 21).^305,306^ This method utilizes a dual-tagging strategy, where a cargo is labeled with both a standard epitope tag (HA) for total protein tracking and an AviTag peptide for cytosolic biotinylation. Upon reaching the cytosol, the AviTag is covalently modified by the BirA enzyme, enabling the parallel determination of total uptake and absolute cytosolic concentration via quantitative Western blotting. Similarly, the CytoSNAP assay employs cell lines expressing a SNAP-tag fusion protein for cytosol-specific detection.^307^ The cargo is labeled with a bifunctional biotin-benzylguanine tag and, upon successful delivery, is covalently captured by the SNAP-tag. Subsequent cell lysis and immunoprecipitation enable a highly sensitive picomolar ELISA-based readout for the absolute quantification of both total and cytosolic concentrations. However, as with all assays that require small-molecule probes or fluorescent markers, potential degradation of the linker and/or the tag itself can introduce quantification artifacts.^308^ This aspect must be carefully assessed, especially when dealing with more challenging conditions present in endolysosomal compartments.^288^

**Figure 21:**
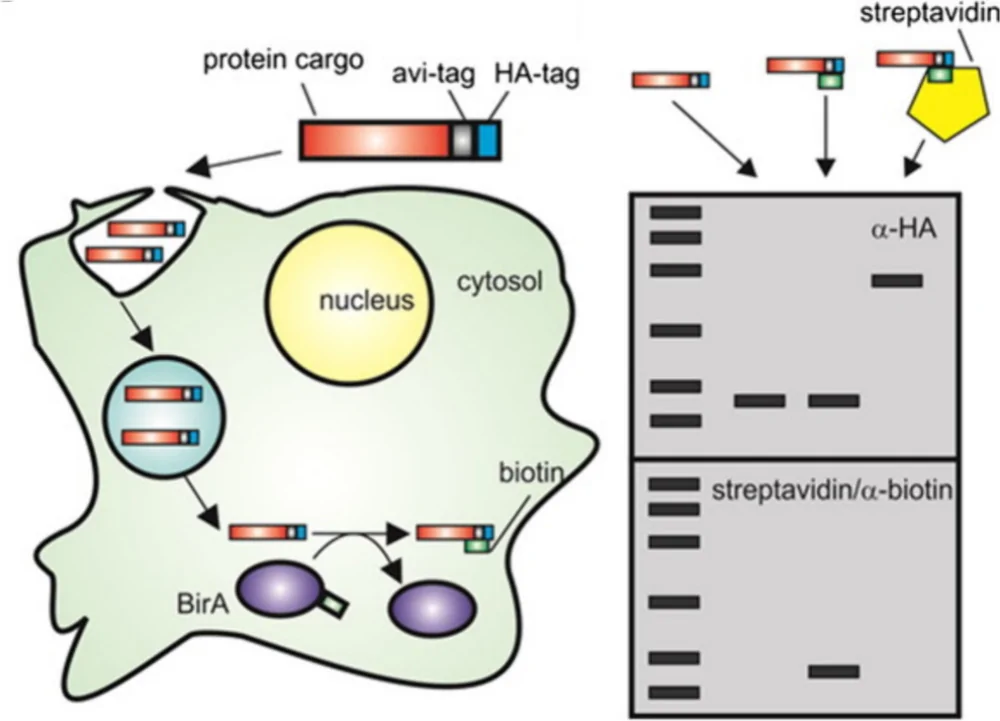
Mechanism of biotin ligase (BirA) assay for absolute quantification of protein delivery efficiency. (left) Western blot-based band intensities are used to quantify the amount of proteins in the cytosol. Adapted with permission from ref 305. Copyright 2015 Elsevier.

In summary, the toolbox for protein delivery is highly diverse, with method selection driven largely by available instrumentation and required sensitivity. Crucially, the choice depends on the specific research objective, whether it is to determine absolute concentration values, endosomal escape efficiencies, or relative delivery trends.

#### 3.3.3 Assessing Functional Delivery of Cargo Proteins

Beyond quantifying the amount of cytosolically delivered protein, it is crucial to confirm that the cargo remains functionally active. Unlike oligonucleotide cargoes, the proper tertiary/quaternary structure of proteins directly relates to their function and must therefore be preserved throughout the entire delivery process, which can be challenging depending on the modification or delivery pathway. To bridge the gap between cytosolic delivery and biological response, a diverse selection of functional reporters is available, as summarized in other reviews.^21,310^

While delivery of GFP-derived fluorescent proteins often serves as an indicator of preserved protein structure, they are relatively robust compared with more sensitive therapeutic cargoes, e.g., enzymes, transcription factors or specific binders. Furthermore, besides cytosolic delivery, many proteins require precise subcellular localization to fulfill their biological function. For example, to achieve targeted delivery to the nucleus, one must either aim for protein size <60 kDa that could translocate through nuclear pores or incorporate a nuclear localization signal (NLS) for active transport through the importin α/β system. One widely used reporter cargo is Cre recombinase, which depends on nuclear localization to elicit a cellular response. Cre is a genome-editing enzyme that allows for gene recombination in reporter cells and a change in fluorescence emission upon successful delivery, which can be easily assessed via microscopy (Figure 22A).^142,167,311,312^ Other prominent examples of genome-editing cargo investigated for delivery include proteins from the Cas family (Cas9, Cas12) with functional readout derived either from deep sequencing or fluorescence microscopy (Figure 22B), similar to the Cre system.^283,313–315^ Transcription factors for cell reprogramming or differentiation were also used as functional cargo with the readout based on the resulting change in cellular phenotype.^311^ Beyond direct genomic modulation, camelid GFP-binding nanobodies have been successfully delivered and shown to maintain their binding affinity to GFP or specific fusion proteins as also confirmed and utilized by super-resolution microscopy.^29,32^ A more therapeutically relevant strategy involves modulating endogenous protein function. For instance, cytosolic delivery of functional nanobodies has been shown to bind and restore the activity of misfolded protein regulators, with success validated by various biochemical assays.^316^ A distinct category of functional protein cargo relies on cell death as a positive readout. Various enzymes, e.g., RNase A, cytochrome c and saporin, have been used to evaluate delivery efficiency via pro-apoptotic activity (Figure 23).^157,283,313–315,317,318^

**Figure 22:**
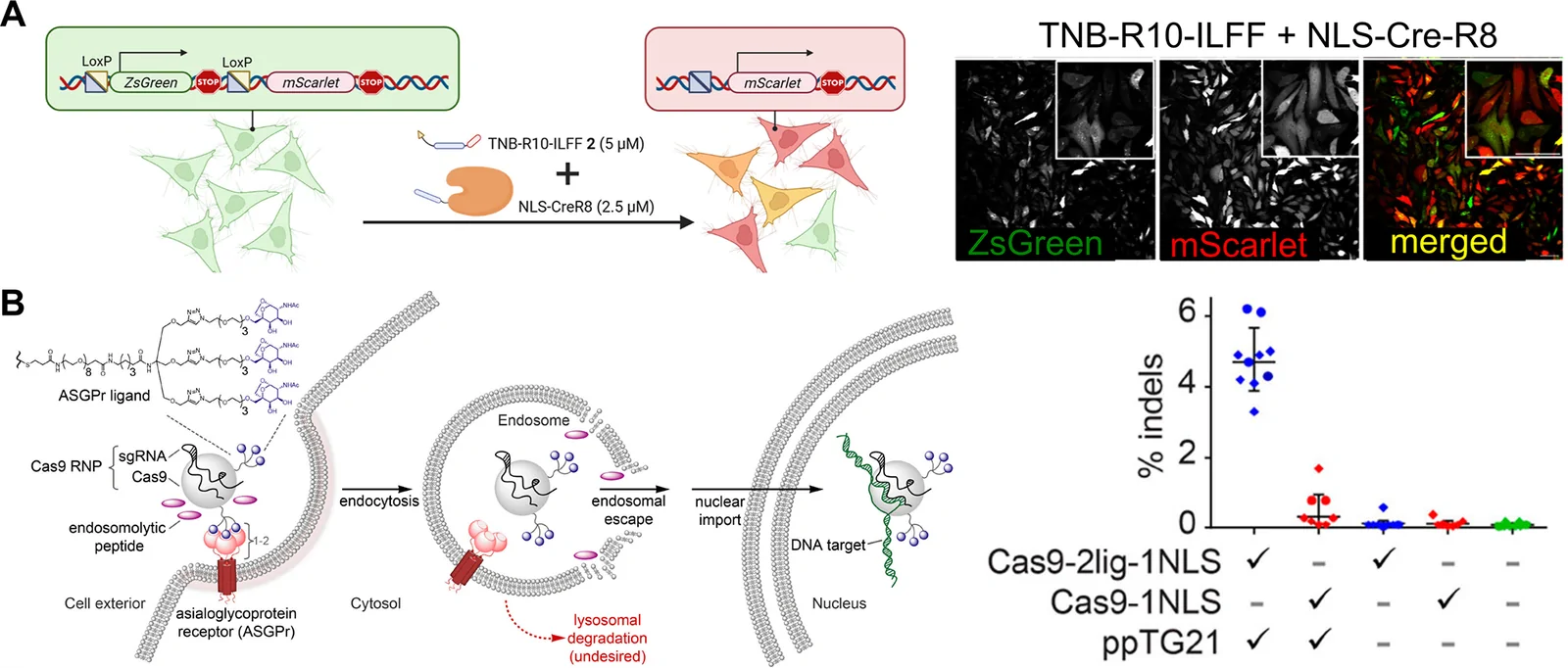
Readout of successful delivery of gene-editing protein (A) Cre recombinase, Reproduced from ref 167. Copyright 2023 American Chemical Society, and (B) CRISPR-Cas9 ribonucleoprotein complex, (B) Reproduced from ref 314. Copyright 2018 American Chemical Society.

**Figure 23:**
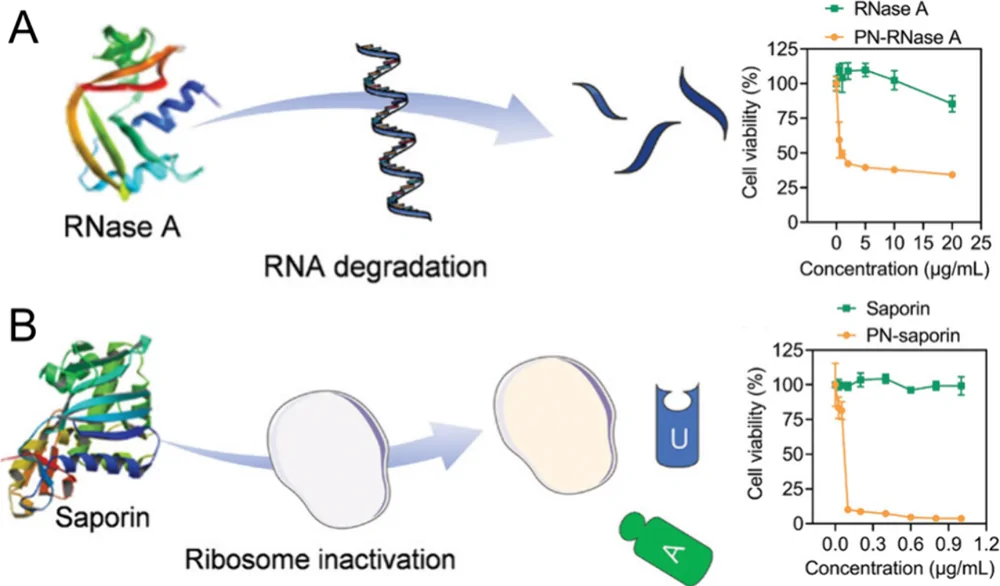
Delivery of toxic protein payloads. (A) RNase A and (B) Saporin delivery using boronic acid-modification. Adapted with permission from ref 283. Copyright 2022 John Wiley and Sons.

One important factor to consider when evaluating functional assays is that for most reported approaches, dose–response relationships remain unknown.^310^ Some assays require only a small amount of functional cargo to be delivered to produce a measurable response, while others may require near-stoichiometric amounts. Therefore, functional readouts should be complemented by quantification assays. In addition, while cell-lethal functional assays might offer a straightforward readout, they necessitate rigorous controls to ensure that the observed cell death is a direct result of the cargo’s biological activity rather than intrinsic toxicity of the delivery vehicle.^319^

#### 3.3.4 Accounting for Cytotoxicity

Successful cellular delivery requires manipulation of either the plasma or the endosomal membrane, a process that can potentially cause irreparable damage to the cell.^319^ Since proteins are large macromolecular cargoes, they require a significant disruption of these biological barriers to access the cytosol. Consequently, a common limitation among delivery methods is the inherent trade-off between delivery efficiency and cytotoxicity.^320^ It is therefore critical to carefully assess the cytotoxicity of protein delivery techniques in cell-based assays for several reasons. First and foremost, these assays are essential for optimizing the composition of delivery vectors to identify the most efficient candidates. Characterizing the relationship between delivery efficiency and cell viability allows for the definition of optimal concentration ranges, ensuring that the vector does not cause cellular damage while still achieving the desired effect. Furthermore, as mentioned above, toxicity profiling is necessary to validate the functional outcomes of toxic cargoes, ensuring that a biological response is the result of delivered protein’s activity rather than an artifact resulting from compromised cellular health. In this section, we highlight the most commonly used assays performed with respect to protein delivery (Table 3) and refer readers to extensive reviews and the list of viability testing methods of cell cultures from the Guidance Document on Good In Vitro Method Practices (GIVIMP) by the Organization for Economic Co-operation and Development (OECD).^321–323^

**Table 3:** Common Cytotoxicity Assays in the Context of Protein Delivery

target	assay type	mechanism	readout	protein cargo
**Membrane Integrity/Necrosis**	LDH Release	Leakage of cytosolic lactate dehydrogenase into the supernatant	Fluorescence	mCherry^157,167^
			Absorbance	Nanobody^316^
			Luminescence	
	Live/Dead	Intracellular esterase activity (live)/DNA staining upon membrane rupture (dead)	Fluorescence	Ubiquitin^327^
	Cell Stain			GFP, Estrogen-Related Receptor β^328^
				
**Apoptosis Markers**	Annexin V/PI	Phosphatidylserine detection (early apoptosis) and PI influx (late apoptosis/secondary necrosis)	Fluorescence	mCherry^167^
				RNase^157^

	Caspase 3/7 Activity	Proteolytic cleavage of specific substrates by executioner caspases	Fluorescence	RNase^157^
			Absorbance	
			Luminescence	

	TUNEL Assay	Enzymatic fluorescent labeling of fragmented DNA (late apoptosis)	Fluorescence	GFP, Estrogen-Related Receptor β^328^

**Metabolic Activity**	ATP Content	Detection of total cellular ATP (energetic stress)	Luminescence	β-Galactosidase^329^
				BSA, RNase, Saporin^283^

	MTT	Intracellular substrate reduction to insoluble formazan crystals by oxidoreductase enzymes	Absorbance	mCherry^329^
				GFP^282^
				RNase^330^

	Resazurin	Reduction of nonfluorescent resazurin dye to fluorescent resorufin by mitochondrial reductase	Fluorescence	GFP^331^
				Affibodies^332^

In the context of protein delivery strategies, it is essential to distinguish between nonselective membrane disruption and broader cytotoxic effects on cell viability. The former can be assessed using membrane integrity assays that detect the leakage of intracellular molecules, such as lactate dehydrogenase (LDH), or the influx of membrane-impermeable dyes such as trypan blue and propidium iodide (PI). In contrast, general cell viability is typically measured by evaluating metabolic activity through the conversion of indicators such as MTT, WST-1, or Resazurin, or by quantifying ATP production (Figure 24). By assessing these factors at early time points (1–2 h post-treatment), researchers can isolate necrosis-related effects caused by membrane damage, while extending the analysis to 24 h allows detection of the combined effects of necrosis and delayed apoptosis. In contrast to necrosis, apoptosis or programmed cell death is a process executed through specific intracellular signaling pathways.^324^ This transition can be monitored by observing morphological changes, such as cell shrinkage and membrane blebbing, or by detecting biochemical markers, such as caspase activation and the translocation of phosphatidylserine to the outer leaflet of the plasma membrane via Annexin V staining.^325^ When cytotoxic proteins (e.g., RNases) are used as functional reporters of delivery, it is essential to perform these specific assays to validate that the observed cell death results from the intended enzymatic or biological activity of the cargo rather than from nonselective membrane damage caused by the delivery vehicle itself. Furthermore, care should be taken to analyze whether specific fragments or chemical components of the respective delivery system might interfere with endogenous cellular processes.

**Figure 24:**
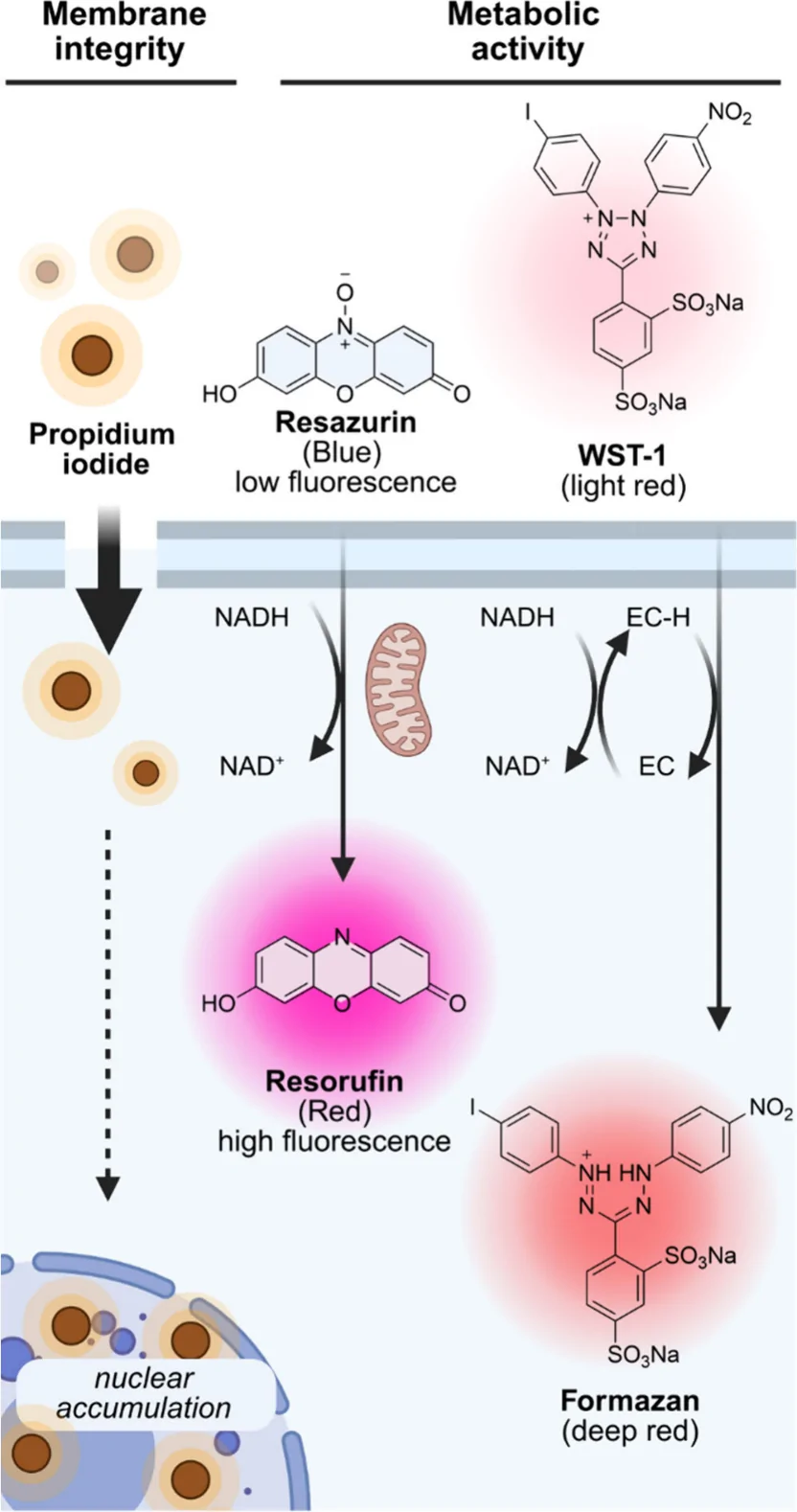
Commonly employed cellular viability assays to assess membrane integrity or metabolic activity. Propidium iodide (PI) enters cells with compromised cellular membrane integrity and accumulates within the nucleus where it intercalates with double-stranded RNA and DNA. Resazurin and WST-1 are both measuring mitochondrial reduction capacity from resorufin and formazan dyes, respectively. Electron coupling reagent (EC). Created with https://BioRender.com.

One essential aspect of accurate cytotoxicity analysis is the establishment of appropriate controls. A comprehensive experimental setup should include the delivery mixture, the protein cargo alone, the vector alone, and vehicle-only controls (e.g., DMSO) to account for potential artifacts introduced during medium exchange or washing steps. Ideally, these controls should be evaluated across a broad concentration range to determine the onset of cellular stress. While employing two orthogonal assays is considered good practice for general cytotoxicity assessment, validating protein-induced apoptosis specifically requires at least two independent, complementary methods. This multiparametric approach ensures that the observed cell death is a direct consequence of the delivered cargo’s intracellular activity rather than an experimental or procedural artifact. Another important consideration when using fluorescently labeled proteins is their potential to interfere with assay readouts, therefore, compatibility has to be ensured. Commercial assays offer diverse readouts, including absorbance, fluorescence, and bioluminescence, which allows for necessary orthogonality to prevent spectral overlap with the cargo. Tailored methods can support multiplexing and provide the high sensitivity needed for high-throughput screening or challenging systems.^326^ Integrating a comprehensive cytotoxicity profile with mechanistic, quantitative, and functional investigations establishes the minimum characterization required to rigorously report and validate any novel protein delivery strategy.

## 4 How Can We Enable Delivery Using Protein Modifications?

Chemical and biochemical modifications of proteins have been investigated to enable cell permeability and allow functional protein delivery. The employed approaches can be categorized into surface grafting, genetic fusion, and bioconjugation strategies (Figure 25). In the following, we will discuss examples, advantages, and limitations of each strategy.

**Figure 25:**
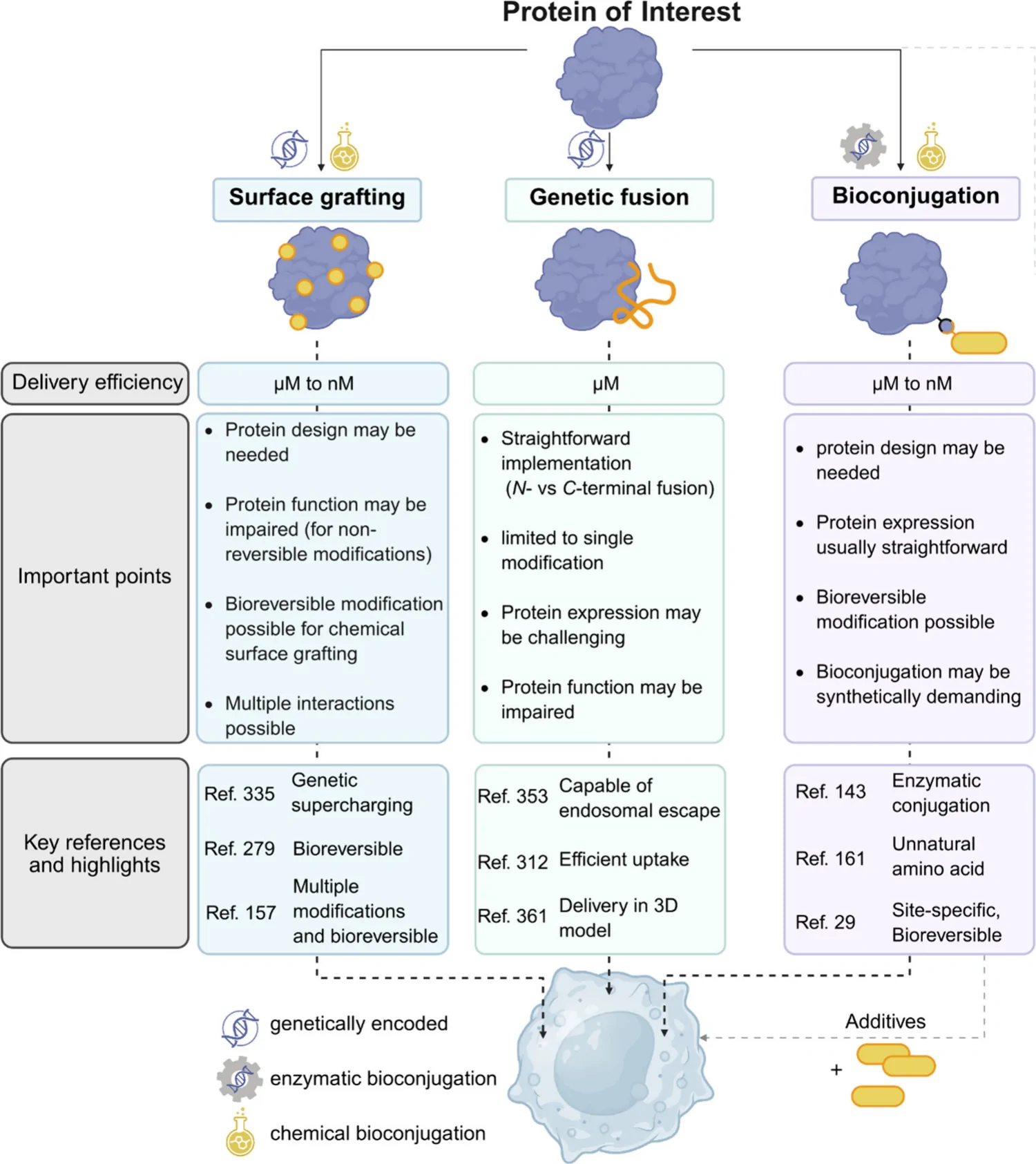
Classification and general considerations of protein modification strategies to confer membrane permeability, including genetic, enzymatic, and chemical approaches. Created with https://BioRender.com.

### 4.1 Surface Grafting

Observing that small arginine-rich cationic peptides are efficiently internalized by cells and that certain ribonucleases with cationic residues display cell permeability,^333,334^ Raines and co-workers studied arginine grafting through site-directed mutagenesis (Figure 26A). As model systems, they chose intrinsically fluorescent GFP and generated a cell-permeable variant by introducing single amino acid mutations to arginine. Incubation of HeLa cells with the modified protein resulted in dose-dependent fluorescence, predominantly confined to vesicles, pointing toward endosomal uptake.^335^ Shortly thereafter, the Liu lab reported the same strategy termed “genetic supercharging”, to increase the physicochemical stability of heavily positively charged (arginine and lysine) and negatively charged (glutamic and aspartic acid) GFP.^336^ Their mechanistic studies of cellular uptake suggest delivery via multiple mechanisms (clathrin-mediated endocytosis rather than macropinocytosis), with significant reliance on binding of the positively charged protein to proteoglycans.^156,285^ By simply mixing the supercharged protein GFP with negatively charged small RNAs and DNA plasmids they demonstrated transfection of respective RNAs and DNA plasmids into human cell lines.^156^ Upon fusion of supercharged GFP with Cre recombinase, they could significantly enhance genetic recombination compared with wild-type protein or Tat and Arg_10_ fusion proteins.^312^ Negatively (super)­charged proteins do not confer cell permeability on their own but are used in combination with positively charged polymers or lipids to form nanoparticles for cellular delivery.^337–340^ The concept of substituting solvent-exposed neutral or negatively charged amino acid residues of proteins, also described as resurfacing, can be applied to the design of proteins (e.g., nanobodies) to endow functional proteins with cell permeability.^341^ Rational approaches can be supported by computational protein design to impart cell-penetrating capabilities on *de novo* designed proteins with specific target-binding motifs.^342,343^

**Figure 26:**
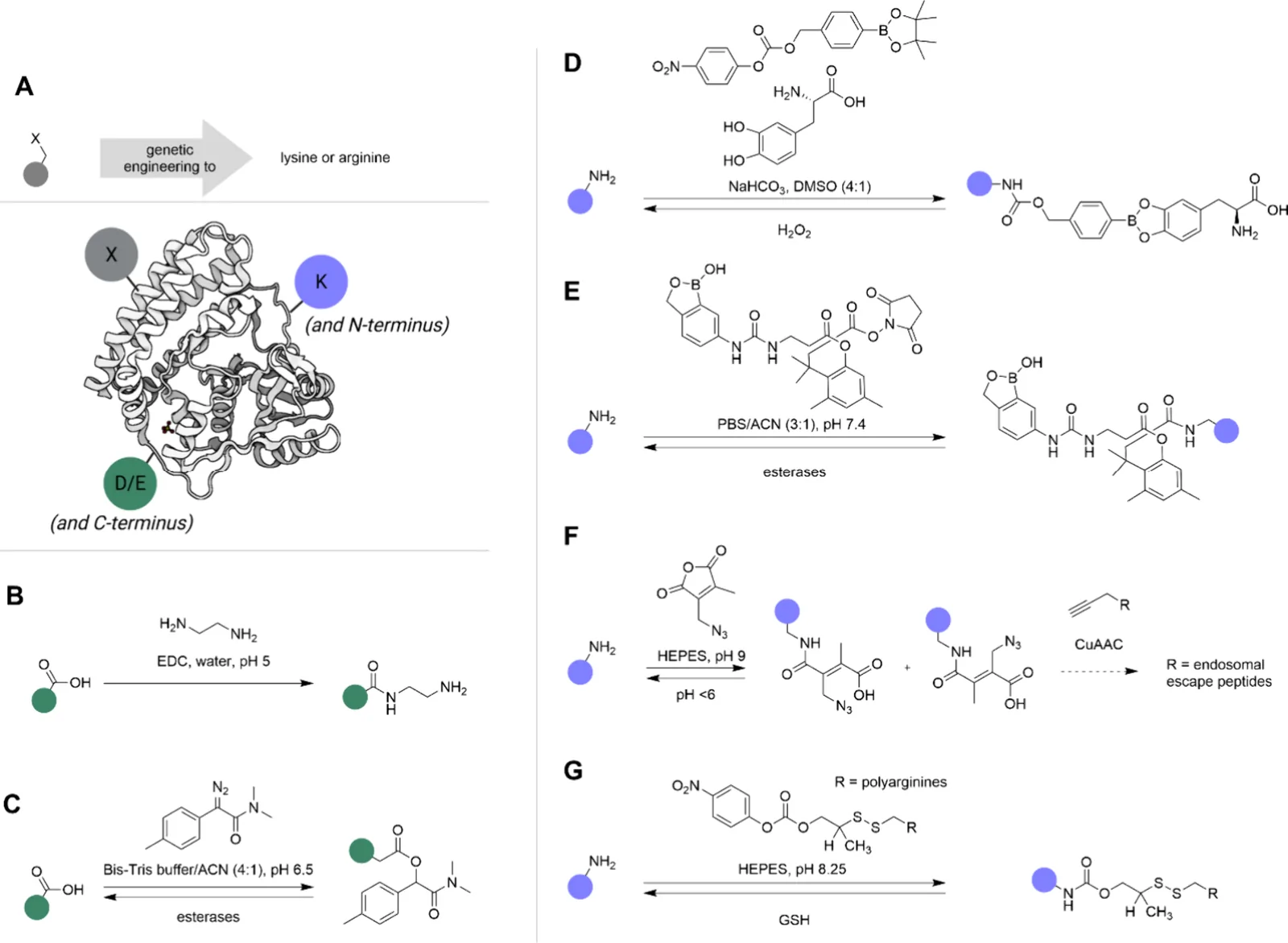
Single-point mutations and chemoselective reagents for protein surface grafting to introduce cell permeability. (A) Genetic grafting of arginine or lysine residues. (B) Permanent cationization of proteins via amid coupling of carboxylic acids with ethylenediamine.^344^ (C) Esterase-cleavable diazo-modification of carboxylic acids.^345,346^ (D) Peroxide-sensitive amine modification enabling l-type amino acid transporter-mediated uptake.^347^ (E) Esterase-cleavable boronic acid modification on amines.^348^ (F) Acid-labile, traceless linker via CuAAC.^282^ (G) Bioreversible arginine modification on amino groups (BioRAM).^157^

Rather than genetic manipulation, functional amino acid side chains can be chemoselectively modified in a heterogeneous manner to confer cell permeability on a protein. The first example was reported by Yamada and Kosaka in 2001, who permanently cationized RNase A/RNase 1 using ethylenediamine and 1-ethyl-3-(-3-(dimethylamino)­propyl)­carbodiimide (EDC) to replace negatively charged carboxyl groups with surface-exposed amines (Figure 26B).^344^ The “chemically supercharged” enzyme showed higher cytotoxicity in cell-based experiments, which the authors attributed to enhanced cell-surface binding and cellular uptake. This observation was followed up by Futami et al., who permanently cationized avidin as a carrier for biotinylated proteins (GFP and simian virus 40 large-T antigen) that were complexed with avidin.^349^ The biotin was connected through a disulfide bridge to the protein, ensuring cytosolic cleavage and release from the avidin complex. Raines and co-workers employed bioreversible esterification of carboxylic acids on proteins using stabilized diazo compounds (Figure 26C).^345,346^ The masking of negatively charged amino acid residues with neutral esters increases the overall positive charge of the protein. The formed esters are cleaved intracellularly by esterases, allowing for traceless delivery of GFP and enzymatically active RNase 1.^279,318^ In a follow-up study, they demonstrated the use of a modular diazo compound with increased intracellular cleavage rates and an additional disulfide conjugation site, which was used to install a cyclic decaarginine (cR_10_) moiety for delivery of GFP and cytochrome *c*.^279,318^

In a different approach, Raines and co-workers demonstrated that EDC-coupling of several boronic acid-containing amine derivatives to aspartic or glutamic acid residues on the protein surface can promote cellular uptake (Figure 26D).^347^ Boronic acids readily form boronate esters with the 1,2- and 1,3-diols of saccharides on the cell surface.^347^ Upon endosomal internalization and endosomal acidification, this interaction is weakened along with decreasing pH, rendering the free boronates more hydrophobic, allowing for translocation into the cytosol. In a second-generation, they introduced an esterase-cleavable boronic acid on protein surface amines using NHS-esters, providing a bioreversible modification (Figure 26E).^348^ Yin and colleagues reported endocytosis-independent delivery of multiple functional proteins, including Cas9, RNase A, and β-galactosidase, by reversible modification of amines with H_2_O_2_-responsive 4-nitrophenyl 4-(4,4,5,5-tetramethyl-1,3,2-dioxaborolan-2-yl) benzyl carbonate and subsequent complexation with 3,4-dihydroxy-l-phenylalanine. They demonstrated selective uptake for such modified proteins in l-type amino acid transporter 1 overexpressing cells, a mechanism previously explored only for small molecules (Figure 26D).^283^

More generally, those concepts demonstrate that a dedicated transduction domain is not a necessary component of a cell-permeable protein but that permeability can be achieved by modifying the protein surface. Introduction of positive surface charge enhances cell–surface interactions and has been successfully applied to promote the cellular uptake of protein cargo.

Further protein delivery methods have evolved based on surface grafting using larger vectors. Wagner et al. have introduced an acid-labile traceless linker for the modification of amines, which is used to install branched transduction oligomers via copper-catalyzed click reaction (Figure 26F).^282^ Through endosomal acidification, the linker is cleaved, thereby facilitating the endosomal release of NLS-EGFP and β-galactosidase into the cytosol of HeLa cells. They have further extended this concept to copper-free click chemistry, with improved oligomers or endosomal-escape peptides to deliver RNase A and to build receptor-targeting, tumor-activated systems.^330,350,351^ By means of bioreversible modification reactions, the Hackenberger Lab introduced heterogeneous modifications of solvent-exposed amines with arginines (BioReversible Arginine Modification, BioRAM) of proteins as an alternative to site-selective conjugation of polyarginines (Figure 26G), further discussed in section 4.3.^157^

Obviously, it is important to ensure that such amino acid modifications are either introduced transiently and removed inside the cell or, if permanent, do not perturb protein function or subcellular localization, especially when using heterogeneous labeling strategies.^352^

### 4.2 Genetic Fusion

Protein transduction domains such as cell-penetrating peptides, endosomolytic peptides, or inherently permeable protein moieties can be employed to render proteins cell permeable.^21^ Due to their peptidic nature, these domains can be conveniently fused to a protein cargo via genetic fusion techniques, resulting in a protein that can be expressed using standard protein expression methods. Early studies applied TAT–protein fusion constructs to investigate *in vivo* delivery in mice.^353^ Dowdy and co-workers reported the uptake of functional β-galactosidase in cultured cells, as well as in multiple organs in mice, by detecting the enzyme’s activity.^353^ Following that proof-of-concept, CPP-fusion proteins have been investigated for various therapeutic applications. An example of potential applications for cancer treatment was reported by Matsui and colleagues.^328,354,355^ They successfully delivered functional p53 protein as a polyarginine-containing R11-p53 fusion protein *in cellulo* as well as in a mouse model and achieved growth inhibition of bladder tumors. The delivery of functional proteins also has great potential for enzyme replacement therapy, as demonstrated by the successful delivery of TAT-fused mitochondrial enzymes into patient cells and into mice possessing mitochondrial disorder lipoamide dehydrogenase deficiency (see section 2.2).^356,357^ Since CPPs have also been shown to cross the blood–brain barrier, their use in protecting neuronal cells against ischemic brain injury by delivery of glyoxalase-CPP and antiapoptotic factor FNK-CPP fusion proteins has been tested *in cellulo* and in gerbil models.^355,358^ Gene therapy machineries can, likewise, be delivered with protein–peptide fusion complexes as demonstrated by Doudna and co-workers.^315^ Another application of CPP-fusion proteins in basic research is the manipulation of human mesenchymal stromal cells as demonstrated by the delivery of an R7-ESRRB fusion protein.^328^ However, utilizing CPP-fusion proteins lacking intracellular cleavage sites has drawbacks. Employing cationic CPPs as delivery vectors can lead to unintended accumulation in cellular compartments, specifically the nucleolus if not cleaved off after delivery, potentially hampering protein function.^29,359^ Despite the impressive laboratory-based results in animal models, only a few CPP-fusion proteins have advanced to clinical trials, and none have yet been approved by the FDA.^130,360^

Peptide-based strategies designed to induce endosomal escape have also been fused to functional proteins. The 30-residue amphipathic peptide GALA and the virus-derived hemagglutinin-2 (HA2) rely on pH-dependent stabilization of their secondary α-helical structure to disrupt lipid bilayers (Figure 27A).^144,168^ Both peptides have been fused to fluorescent proteins and showed cytosolic delivery into model cells.^168^ In a similar manner, lipid composition-sensitive endosomolytic peptides (e.g., L17E and L17ER4) have been fused to Cre recombinase and showed successful uptake in cells and in mouse models.^146^ A crucial issue with fusing an endosomolytic sequence is the reduced expression of the fusion proteins. Futaki and co-workers attribute this to membrane-lytic peptides rupturing bacteria during expression. Hence, they propose relying instead on post-translational modification with the delivery vector L17ER4 (Figure 27B).^146^

**Figure 27:**
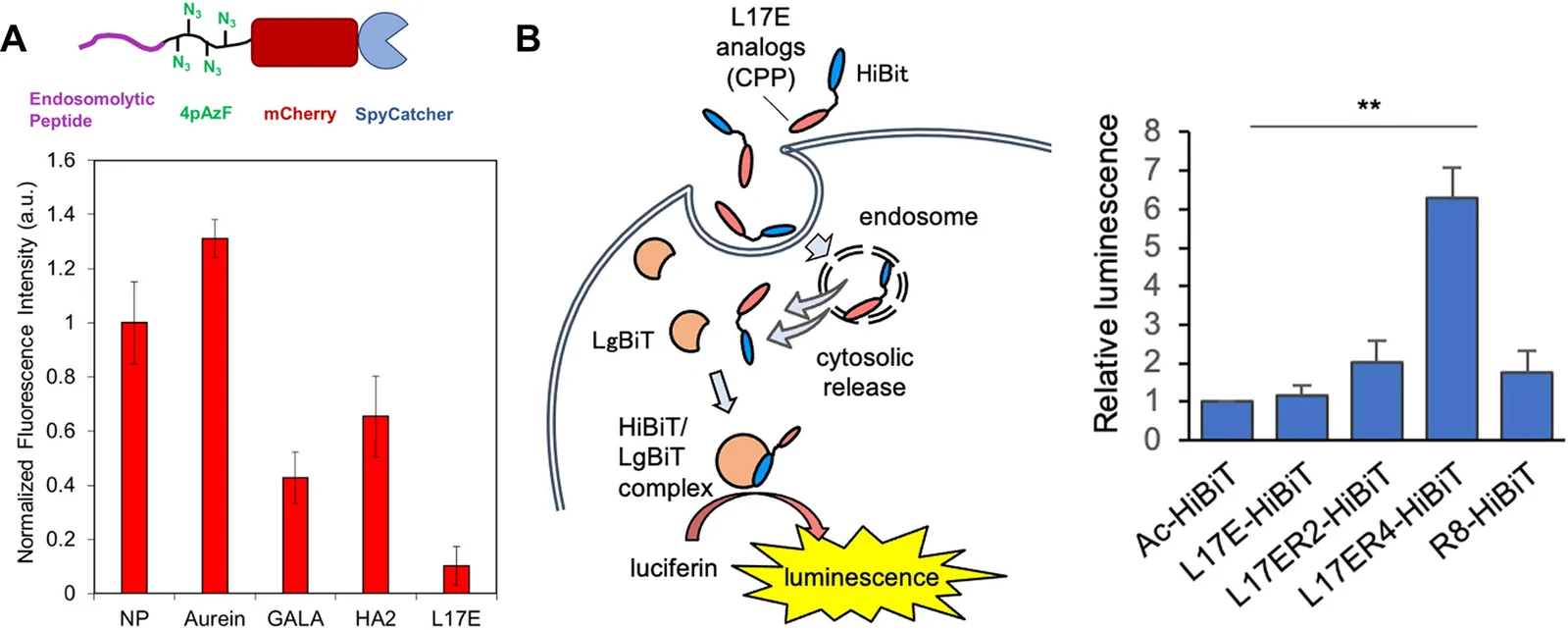
Representative data showing the delivery of genetically fused protein delivery vectors. (A) fusion protein of an endosomolytic peptide and mCherry. Reproduced from ref 168. Copyright 2022 American Chemical Society. (B) L17E-fused split luciferase. Adapted with permission from ref 146. Copyright 2022 Elsevier.

Beyond linear peptides, cell-penetrating protein moieties can also be fused to a functional protein of interest to enable the protein delivery. The aforementioned supercharged GFP­(36+), introduced by the Liu group, has been fused to proteins and has shown functional delivery of ubiquitin and Cre recombinase.^285,312^ Similarly, a GFP containing 29 His residues was investigated by Yao et al. as a delivery vector for proteins to selectively permeate tumor cells in a pH-dependent manner (Figure 28).^361^ The group used their His_29_GFP to deliver a fluorescent model protein and functional RNase into cells and a 3D tumor model.^361^

**Figure 28:**
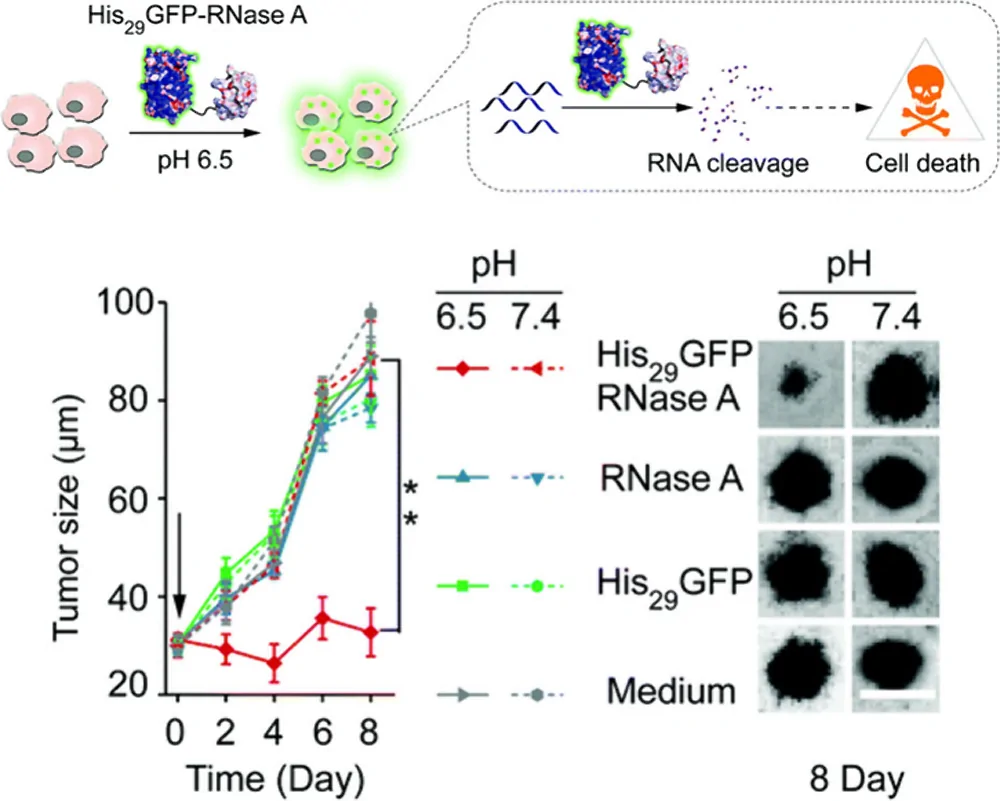
Tumor delivery of cytotoxic RNase fused with GFP having 29 residues of histidine (His_29_-GFP-RNaseA). Adapted with permission from ref 360. Copyright 2018 Royal Society of Chemistry.

Another prominent protein used for protein delivery is the ZF5.3 miniature protein developed by the Schepartz group.^362^ Upon fusing ZF5.3 to the *N*- or *C*-terminus of a protein cargo, the fusion protein is taken up by cells via the endosomal pathway, where pH-dependent unfolding of ZF5.3 induces endosomal escape.^363^ Compared to several endosomolytic peptides, ZF5.3 has demonstrated improved efficiency^291^ and has been applicable for *in cellulo* and *in vivo* delivery of functional, therapeutically relevant enzymes.^290^ The permeable miniature protein has also been adopted by other groups, including for the generation of a nanobody-based ZF5.3 fusion protein for the targeted degradation of BCL11A in cell lines and primary cells.^290^

Although it has not been systematically evaluated, one can envision that the fusion of a cell-permeable protein to a protein cargo might have disadvantages for bacterial expression, especially since lower yields have been reported for certain endosomolytic delivery vector fusions, as discussed before.^168,364^ However, genetic fusion strategies, regardless of the cell delivery entity, commonly rely on fusion at the *N*- or *C*-terminus of the protein cargo. This restricts the position of the delivery vector and is not beneficial when a free terminus is required for protein function.^30,290,365^ Alternative strategies, such as the insertion of CPPs into an exposed loop region of the protein to increase protein permeability, have been published;^366^ however, this strategy is dependent on individual protein geometry and has found limited further use until now.

### 4.3 Bioconjugation

With the advancement of chemoselective or bioorthogonal reactions, chemical modification of protein cargos with delivery vectors has become an important strategy in engineering cell-permeable proteins.^367^ The chemical or chemoenzymatic bioconjugation of delivery vectors to protein cargoes holds several advantages.

Besides fine-tuning the amount and specific placement, bioconjugation allows for the attachment of chemically modified delivery vectors, as opposed to genetic fusion strategies that are restricted to natural amino acids, as discussed in the previous section. Hence, bioconjugation can leverage optimized CPPs with improved delivery performance.^130,262,368^ In such peptidic delivery vectors, noncanonical amino acids with unnatural side chains, backbone modifications, or d-amino acids can be incorporated.^369,370^ Additionally, the use of *N*-methylated amino acids^371^ or peptoids^372^ was demonstrated to improve membrane permeability. Powerful delivery vectors have also been reported to contain rigidified peptide backbones or preorganized scaffolds, such as amphipathic polyproline II helix-derived peptides^373^ or cyclic CPPs, for which several protein conjugates are discussed.^374^ Importantly, these modified peptides also exhibit improved stability against proteolytic cleavage.^375^

Chemical protein modification can also generate bioconjugates with cleavable linkages, for instance, disulfides or protease cleavage sites, which enable the intracellular cleavage of the delivery vector.^376^ Finally, protein bioconjugates can be easily equipped with diverse functional modules such as fluorophores, localization tags, cross-linkers, or payloads for additional experimental readouts.

This Review will focus on bioconjugation approaches that have enabled protein delivery. For a more thorough discussion on related chemical aspects and synthetic strategies to obtain these bioconjugates, we refer the reader to dedicated reviews on this topic.^352,377^

#### 4.3.1 Early Examples of Cell-Permeable Protein-Bioconjugates

A very early mention of using bioconjugate delivery vectors to enhance intracellular protein delivery was reported in 1978 by Shen and Ryser.^381^ They utilized carbodiimide-catalyzed coupling^378^ of a polylysine polymer to radiolabeled human serum albumin or to horseradish peroxidase (HRP). The authors report that delivered HRP remained enzymatically active following uptake, concluding that carboxyl conjugation of the polylysine does not impair the activity of the delivered proteins; however, excessive modifications were noted to potentially alter the activity of the protein cargo of interest (Figure 29A). This aspect was confirmed in a later study, in which high loadings of TAT peptides onto antibody Fab fragments increased protein uptake at the expense of their binding activity.^382^ Barsoum and co-workers reported the chemical conjugation of functional proteins with TAT peptides using heterobifunctional cross-linkers to lysine residues on proteins (Figure 29B).^317^ A repertoire of TAT-conjugated proteins was tested for delivery in mice, including β-galactosidase, HRP, RNase A, and domain III of *Pseudomonas* exotoxin A, positioning this and a later similar work for *in vivo* applications.^383^ Besides the promise of early successes, it should be noted that despite numerous clinical evaluations, no cell-permeable protein conjugate has received regulatory approval.

**Figure 29:**
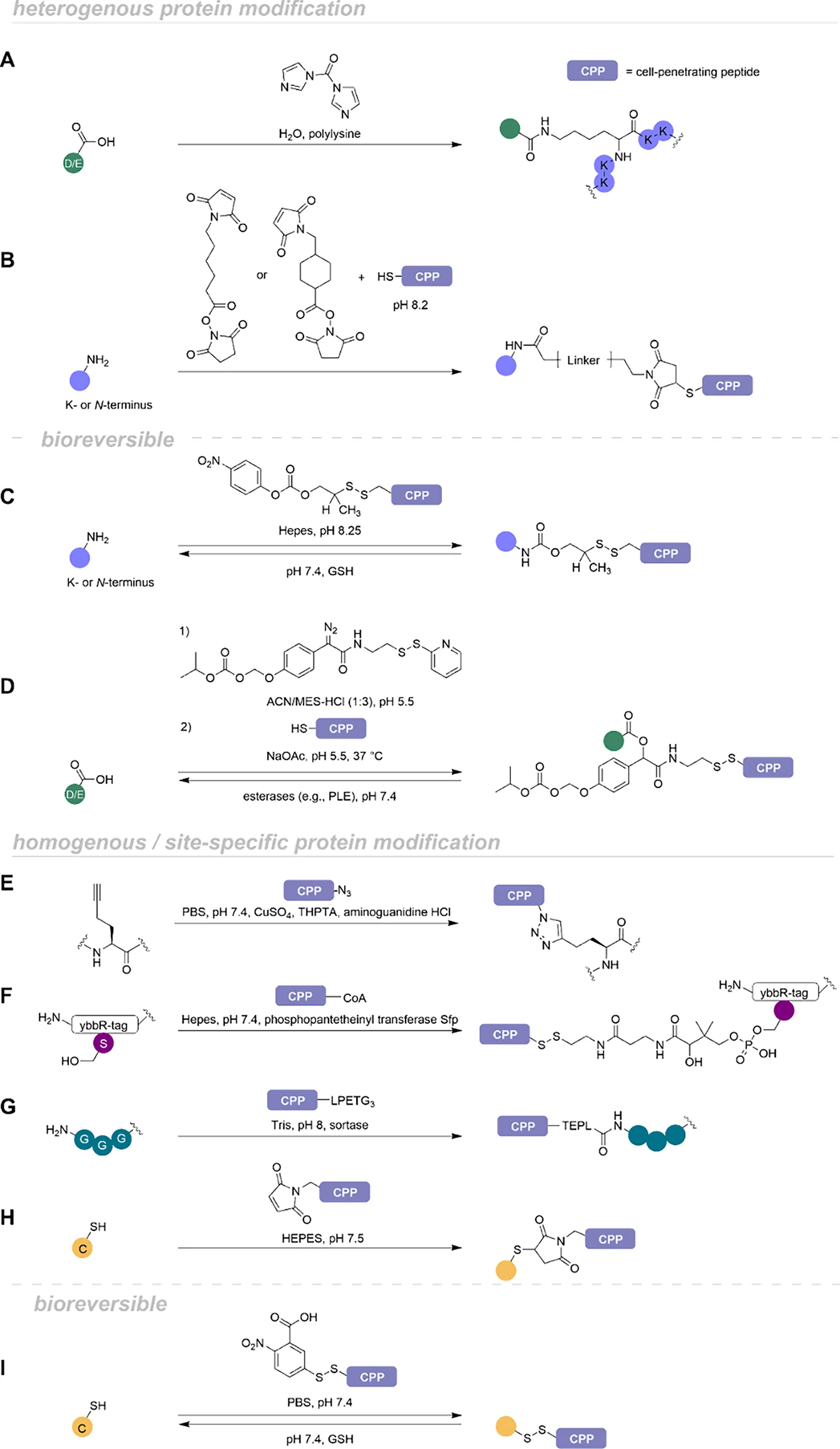
Bioconjugation strategies for the attachment of CPPs to proteins, categorized by site-selectivity of attachment and bioreversibility. (A) Activation of protein-carboxylic acids for polylysine cross-linking.^378^ (B) Bifunctional cross-linkers to connect lysine residues to thiol-containing CPPs.^317^ (C) Bioreversible arginine modification of amino groups.^157^ (D) Modular diazo compound for esterase-cleavable late-stage modification.^379^ (E) Sortase-mediated N-terminal modification.^291^ (F) *N*-terminal modification by phosphopantetheinyltransferase (Sfp).^380^ (G) CuAAC of alkyne-containing amino acids introduced via unnatural protein expression.^281^ (H) Thiol-alkylation of reduced endogenous disulfides or genetically engineered cysteine residues.^359^ (I) Bioreversible disulfide bond formation of reduced endogenous disulfides or genetically engineered cysteine residues.^359^

#### 4.3.2 Protein Conjugates with Cyclic CPPs

Side-chain cyclized arginine-rich CPPs were shown to display immediate bioavailability in contrast to their linear analogs, demonstrating a great opportunity to bypass endosomal pathways.^374^ Subsequently, Cardoso and Hackenberger reported in 2015 that covalent conjugation of cyclized TAT-peptide (cTAT) to GFP using copper-catalyzed click chemistry (CuAAC) enabled the immediate uptake of the protein conjugate through direct translocation at a 50–100 μM concentration.^281^ The groups extended this concept to cyclic decaarginines (cR10) and demonstrated the delivery of nanobodies for intracellular targeting^29^ and cleavable conjugates with distinct subcellular localization (Figure 29E).^359,384^ Higher protein delivery efficacy of cTAT and cR10 compared with their linear counterparts was further validated by independent researchers.^331^ The Brik group demonstrated the use of cR10 to deliver synthetic ubiquitin to study the intracellular role of ubiquitin and ubiquitin-like modifiers and improved delivery efficacy by attachment of a DABCYL derivative, which was also reported by Yousef et al.,^47,327,385^ following up on the work delivering TAT-ubiquitin as a fusion protein^386^ and dimeric, semisynthetic di-TAT.^387^

In parallel, research groups have explored chemical conjugation of cyclic peptides for endosomal escape to overcome the bottleneck of endosomal entrapment. The Pei lab reported the development of synthetic cyclic CPPs, specifically, cFΦR_4_, for improved endosomal escape and cellular delivery of protein.^380^ The group proposes endosomal budding and collapse as the primary means of endosomal escape using their peptides.^380,388^ The disulfide-cleavable cFΦR_4_ peptide was enzymatically conjugated to GFP using phosphopantetheinyl transferase, whereas a noncleavable variant was conjugated to protein tyrosine phosphatase 1B (PTP1B) via a stable thioether linkage (Figure 29F). The GFP conjugate demonstrated cellular uptake by fluorescence microscopy, while the PTP1B conjugate showed intracellular activity. The Schepartz group also utilized sortase ligation to conjugate CPP9 and CPP12, developed by the Pei group, onto the *N*-terminal SNAPtag protein (Figure 29G).^143,291^ Although not delivering an intracellularly functional protein, this report used this construct to quantify proteins that undergo endosomal escape in comparison to Schepartz miniature proteins (see section 4.2).

#### 4.3.3 Thiol-Mediated Uptake

In addition to demonstrating efficiency in endosomal escape, other groups have sought to bypass endosomal uptake altogether by using cell–surface thiols. Various laboratories have used the concept of thiol-mediated uptake since 1990.^389,390^ Matile and co-workers established thiol-mediated uptake, an entry process in which substrates, such as cell-penetrating poly­(disulfide)­s (CPDs) or cyclic oligochalcogenides (COCs), engage in dynamic exchange cascades with exofacial disulfides, thereby forming and reshuffling covalent links to the cell surface during translocation.^198^ For a general introduction, we recommend extensive reviews elsewhere.^214,215,390^

With respect to protein delivery, Yao et al. demonstrated the use of thiol-mediated uptake for the delivery of fluorescently labeled proteins, either by linking CPDs covalently (using homogeneous NHS-labeling or site-selective cysteine conjugation followed by bioorthogonal tetrazine labeling) or noncovalently (employing complexation through avidin/biotin interaction or nitrilotriacetic acid (NTA)/His-tag interaction) to the protein of interest (Figure 30A).^391^ CPDs are polymerized synthetic mimics of polyarginine CPPs, in which the polypeptide backbone was replaced with poly­(disulfide)­s. Upon cellular uptake, CPDs are reduced and depolymerized by cytosolic glutathione.^392^ This concept was further extended by Matile and co-workers to generate cell-penetrating streptavidin (CPS), which is covalently modified with cyclic oligochalcogenides (COCs) (Figure 30B).^393^ CPS tetramers served as carriers for diverse complexed biotinylated cargos, including fluorophores, peptide nucleic acids (PNAs), and nanobodies, through noncovalent complexation and intracellular dissociation through the addition of (desthio)­biotin. The same laboratory also directly modified GFP with cyclic disulfides as cascade exchangers (CAX) (Figure 30C).^394^ The bioreversible esterification, introduced by Raines and co-workers,^279^ was used to functionalize carboxylic acids with azides, which were subsequently used for the conjugation of CAX derived from asparagusic acid via CuAAC. Modified GFP entered multiple cell lines at 20 μM and demonstrated superior tissue penetration, as demonstrated for spheroids. More recently, the same group introduced traceless “CPD grafting-to” thiol conjugation with conjugation rates similar to heteroaromatic sulfones while requiring as little as 50 nM of protein. The resulting grafted peptides and proteins, including antibodies, have been shown to enter cells via TMU and remain functional within the cell.^395^

**Figure 30:**
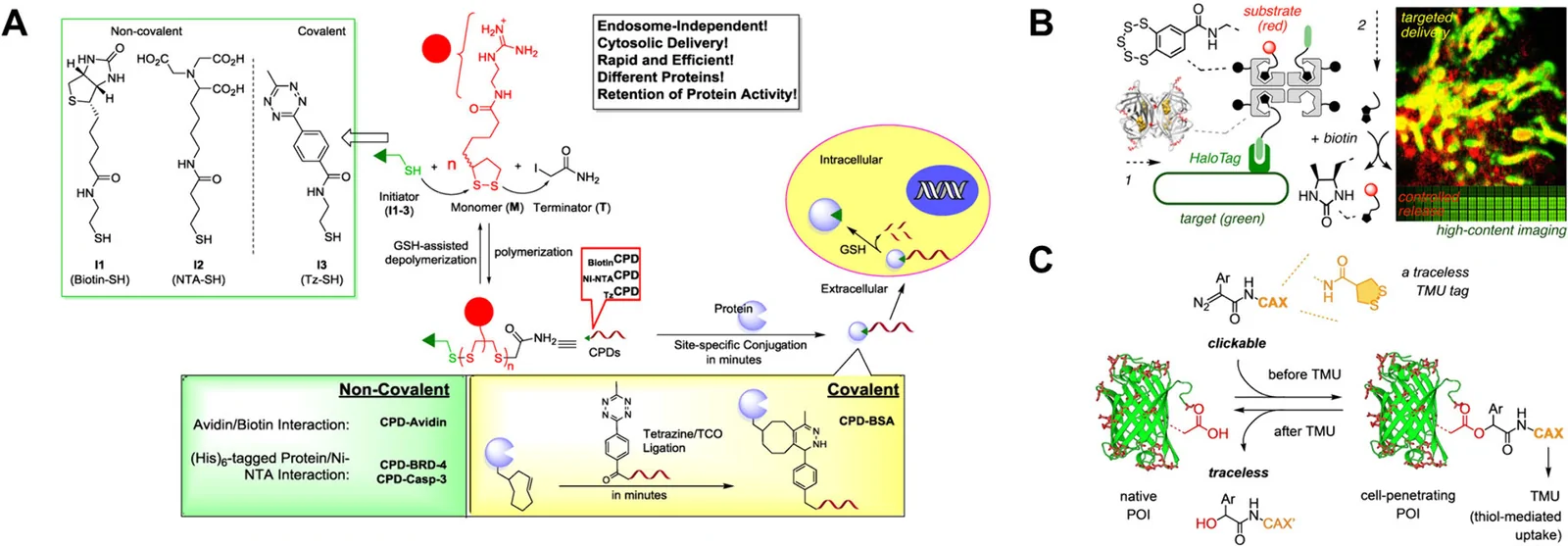
Selected examples for protein delivery using thiol-mediated uptake. (A) Generation of Cell-Penetrating Poly­(disulfide)­s (CPDs) through different initiators to facilitate delivery of protein cargos by covalent and noncovalent modification Reproduced from ref 391. Copyright 2015 American Chemical Society. (B) Cell-penetrating streptavidin modified with benzopolysulfanes on lysine residues, providing a modular scaffold for targeted delivery of substrates, including nanobodies. Reproduced from ref 393. Copyright 2020 American Chemical Society. C) Traceless modification using bioreversible esterification of carboxylic acids to conjugate cascade exchangers (CAX) for the delivery of fluorescent BSA and GFP. Reproduced with permission from ref 394. Copyright 2022 The Author(s), Creative Commons CC-BY license.

#### 4.3.4 CPP-Additives for Direct Translocation

While using higher concentrations of protein conjugates might be feasible for *in cellulo* experiments, it severely limits the practical applicability *in vivo*, with respect to off-target reactions, toxicity, and production. Emerging strategies, discussed in a recent review,^164^ have addressed this limitation by locally enriching CPPs at the plasma or endosomal membrane through multimerization, clustering, coacervate formation, or codelivery, enabling efficient protein transport at low micromolar concentrations. One example for multimerization has been shown by Raines and co-workers, utilizing cR10 in combination with bioreversible protein modification and demonstrating efficient uptake for multiply modified GFP and functional cytochrome *c* even at low micromolar concentrations (Figure 6D).^379^ Although multiple attachments of CPPs decrease the effective concentration, we want to emphasize at this point that CPPs, particularly multivalently presented and cyclic variants, should be carefully assessed for potential toxicity.^164,396^

Another approach to enabling efficient direct translocation is through the use of additives in protein delivery. The groups of Matile and Futaki presented the use of PyB as an additive to deliver GFP-R8.^245^ Initially believed to only work as a counterion to enhance the amphiphilicity of cationic peptides,^397^ PyB was eventually found to affect the membrane dynamics by loosening the membrane packing of the cell membrane.^238,243,398^ As an alternative to small-molecule additives, Nau and co-workers introduced globular dodecaborate superchaotropic clusters as membrane carriers for the delivery of hydrophilic cargoes (i.e., phalloidin and protamine) through the hydrophobic membrane barrier. These boron clusters work by associating with the hydrophilic cargo molecule and stripping away the surrounding water (desolvation). This interaction minimizes the repulsion between the cargo and the lipid membrane barrier, enabling the partitioning of the cargo directly through the membrane via the enthalpy-driven chaotropic effect (the interaction of superchaotropes with hydrophobic phases), as opposed to the entropy-driven hydrophobic effects exploited by counterions such as PyB (Figure 31).^399^

**Figure 31:**
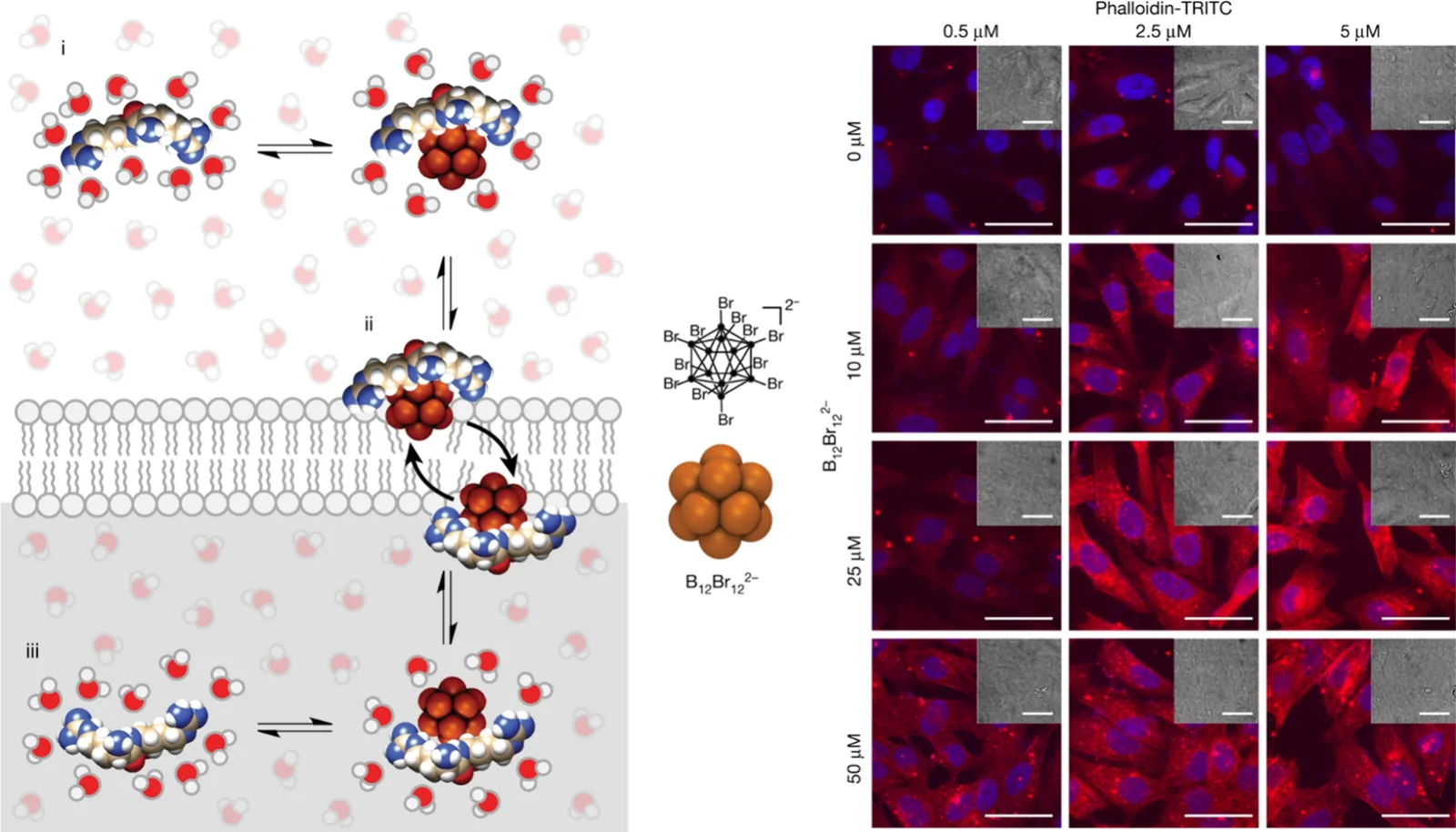
Mechanism of membrane translocation by superchaotropic clusters. (i) Hydrophilic molecules having a high barrier against desolvation and are unable to penetrate the cell membrane. (ii) Enthalpy-driven chaotropic interactions drive desolvation of the cargo and facilitate cargo membrane partitioning and direct translocation. (iii) The chaotropic interaction is reversible, hence dissociation of the complex and release of the cargo inside the cell. (left panel) Representative data of cellular delivery of phalloidin-TRITC using dodecaborate (B_12_X_12_^2–^) superchaotropic clusters (X = H, Cl, Br, and I). (right panel) Reproduced with permission from ref 399. Copyright 2022 The Author(s), Creative Commons CC-BY license.

An early example of a peptide-based additive to enhance direct translocation was mentioned by Hirose et al., where a dodecaarginine (R12) fused with the hydrophobic HAtag peptide, which enhanced the uptake of the peptidic R4-Alexa488 probe.^400^ The Hackenberger lab has approached the problem from a different angle by combining CPP-modified proteins with CPP additives to overcome the concentration limitation for the delivery of CPP-protein conjugates via direct translocation (Figure 32A). This method uses significantly lower CPP-protein cargo concentrations and offers more control over protein delivery, and a linear relationship between the amount of cargo added and quantified signal was observed. The initial finding reported a decaarginine sequence with an *N*-terminal thionitrobenzoic acid disulfide to impart the CPP additives onto the cell surface by a reversible, covalent reaction with exposed thiol groups.^34^ These CPP additives formed nucleation zones, previously reported in the CPP literature, and were hypothesized to provide entry points for CPPs.^155,401^ Independent research groups have picked up CPP additive-mediated delivery for the delivery of a ferroptosis-inducing nanobody^402^ and for high-contrast live-cell imaging of postsynaptic structures.^403^

**Figure 32:**
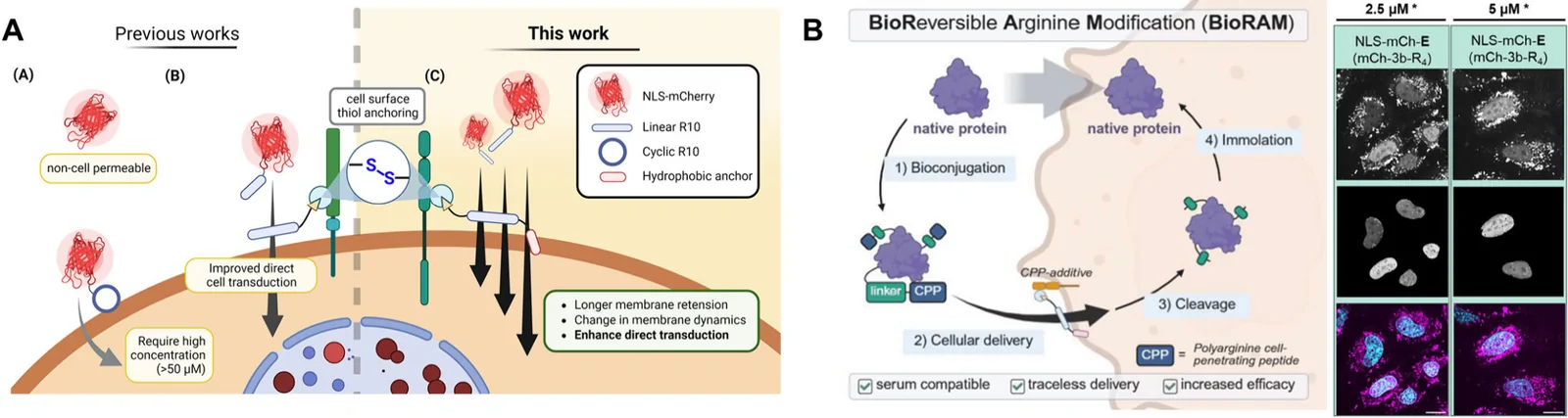
Intracellular delivery using CPP-additives. (A) Second generation of cell-surface retained CPP-additives for the delivery of NLS-mCherry employing a thiol-reactive headgroup and a hydrophobic anchor. Reproduced from ref 167. Copyright 2023 American Chemical Society. (B) Reversible modification of protein cell surface amines (BioRAM) and delivery of NLS-mCherry in HeLa cells by CPP-additives. Reproduced with permission from ref 157. Copyright 2022 The Author(s), Creative Commons CC-BY license.

The CPP-additive design was improved within a second generation by including a hydrophobic peptide anchor (-ILFF) on the *C*-terminus of the CPP-additive, which led to prolonged surface retention and decreased membrane tension, facilitating uptake of NLS-mCherry-R_10_ and enzymatically active Cre-R_8_.^167^ This is supported by other works that also mentioned the notable improvement provided by hydrophobic tags for protein delivery.^400,404–406^ Additionally, CPP-additives carrying polyfluoroalkyl tags were shown to improve protein delivery and to exhibit monomeric lipid insertion in model membranes.^407^ A further advancement of the CPP-additive method was the bioconjugation strategy of CPPs to the protein cargo called BioReversible Arginine Modification (BioRAM) (Figure 32B).^157^ Instead of site-specific modification, the method enabled heterogeneous modification of native proteins on primary amines without the need for genetic engineering and site-selective conjugation and furthermore increased the delivery efficacy of functional protein (RNase A) in the nanomolar range (EC_50_ = 13.5 nM). An optimized disulfide linker ensures intracellular cleavage and complete immolation of the modification to release the native protein. They demonstrated that shortening the linear polyarginine peptide, which is heterogeneously conjugated to the protein, from decaarginines (R_10_) to tetraarginines (R_4_) maintained superior delivery efficacy compared to the site-selective, single-conjugated variant, reminiscent of the chemical supercharging described in section 4.1. Recently, this method has also been expanded to deliver an engineered nanobody to control opioid receptor function.^40^

Lately, Hackenberger et al. reported experimental evidence toward a mechanistic understanding of CPP-additive-mediated protein uptake and elucidated the interplay among key parameters that enable the direct translocation of CPP–protein cargos.^137^ Previously, mechanistic models for direct translocation of CPPs, such as the inverted micelle model, barrel-stave or toroidal pore formation, the carpet model or vesicle budding and collapse have only been demonstrated on a peptide level. All of them involve membrane rearrangement or pore formation processes, which are strongly influenced by the properties of the type and concentration of vector (CPP). Now it was demonstrated that protein entry can occur through membrane potential-driven water pores.^137^ These pores arise from the accumulation of CPP-additives within nucleation zones. Further, the study demonstrated that modification of protein with positively charged amino acids is required for efficient direct translocation, as the resulting driving force along the transmembrane electric field pulls the cargo through the water pore. Direct translocation occurs rapidly, within 30–300 s after CPP application, and is subsequently followed by endosomal uptake of remaining CPP-additives or cargos. Complete depolarization of the membrane fully inhibits direct translocation and establishes the membrane potential as the main driving force for protein uptake, validating previous hypotheses of CPP-induced water pore formation^115,134,251,408,409^ and linking this mechanism to the nucleation zones first described by Brock et al.^155^

During the writing of this Review, a publication by Trofimenko et al. reported an electrophysiological approach to evaluate the internalization of CPPs and homeoproteins. While this article gives further evidence of the involvement of membrane potential, it also mentions the requirement of membrane GAGs in direct translocation across the cell membrane.^410^

## 5 Future Challenges, Perspectives, and Conclusions

As shown in this Review, cellular protein delivery is a vibrant research field that has benefited from contributions from all scientific disciplines in molecular life sciences. Progress, especially in basic research, is further accelerated by meticulous mechanistic investigations and the development of sophisticated delivery strategies, which are bolstered by advances in the scalable production of modified proteins.

From a therapeutic perspective, protein delivery is still predominantly confined to preclinical stages irrespective of the delivery strategy employed. Although CPPs generated considerable excitement upon their discovery 35 years ago, the poor translation of *in vitro* results to clinical settings left many researchers disappointed.^130,411,412^ Recently, the first CPP-containing neurotoxin formulation, based on protein botulinum toxin type A, received FDA approval, a milestone we view as heralding further examples of successfully delivered intracellular proteins.^75^

Nevertheless, further challenges arise in exploiting the full pharmacological potential of cell-permeable protein modalities. Despite growing acceptance of parenteral therapies, evident in the success of long-acting GLP-1 injectables, oral administration remains the preferred mode. Consequently, the gastrointestinal tract poses a formidable challenge to protein stability, necessitating strategies to enhance their resilience.^413^ Here, advancements in peptide science that enable oral bioavailability and extended plasma half-lives may prove transferable to proteins. Lessons can also be drawn from extraordinarily stable natural proteins and advanced biopharmaceuticals as well as emerging peptide stabilization strategies.^414^

Furthermore, protein modification remains labor-intensive, with limited generalizability across targets. More versatile approaches may emerge from formulation strategies or nanocarriers that operate orthogonally to protein-level modifications, offering broader applicability.^414,415^ Alternatively, local delivery, particularly via transmucosal routes, presents an attractive option for existing protein therapeutics and chemically modified variants, facilitating rapid absorption.^76^

Immunogenicity is a critical issue that must be addressed to realize protein delivery for pharmaceutical applications. This is most apparent when cationic lipid nanocarriers are used to deliver macromolecules in *in* *vivo* animal models.^416^ Cationic delivery vectors, ranging from peptides to polymer vectors, are found to interact with antigen-presenting cells and macrophages, thereby eliciting an immune response in mice.^417–419^ On one hand, an immune response may be favored when one intends the vector to also act as an adjuvant. However, special care should be taken when the desired response is not immunological in nature, such as in delivering toxic payloads. Peptide-based delivery systems and drug conjugates are reported to have good delivery efficiencies, to be less immunogenic than conventional lipid nanoparticles, and to be good alternatives.^420–422^ Xiao et al. provide an insightful review that discusses the successful applications of peptide-based modifications and delivery for diseases.^423^

Another critical factor for clinical success is increasing the protein half-life and improving pharmacological robustness.^424^ Chemical strategies, including PEGylation, glycoengineering, lipidation, and albumin fusion, could help increase the size of the protein of interest and prevent renal filtration.^425^ Moreover, some of these modifications were demonstrated to also help prevent proteolysis and adverse immune responses. Despite the promising results, the extensive modification of proteins must be carried out carefully to ensure homogeneity and integrity of the cargo.^426^

Looking ahead, we anticipate further breakthroughs in the field, fueled by recent discoveries and research. First, the adoption of physiologically relevant cell culture models, such as advanced 3D cultures and organoids, will be instrumental in bridging the gap between *in vitro* experiments and *in vivo* studies.^427–430^ Early evaluation of protein delivery strategies in these systems will provide critical insights into the penetration depth and stability under more biomimetic conditions, thereby reducing translational failures and accelerating the identification of viable approaches.

Second, computer-based methods and artificial intelligence (AI) hold promise for further revolutionizing the field of computational protein design.^431–433^ The ability to engineer proteins for virtually any target or function is poised to transform scientific and industrial sectors, including drug development, nanotechnology, and environmental sustainability. Reaching the cytosol remains a primary challenge for proteins to reach their sites of action within the cell. To address this, rational design is being used to engineer cell permeability directly into synthetic structures, such as dual-function *de novo* peptides that combine cell-penetrating capabilities with specific target-binding motifs.^342,343^ We expect continued progress in this area through further advances in our understanding of translocation mechanisms and the physicochemical parameters that influence delivery. Finally, we anticipate that the continued integration of organic synthesis with biochemical and cell biology techniques will enable the targeting of previously undruggable intracellular pathways, not limited to protein–protein interactions but extending to RNA, DNA, and enzymatic activities (e.g., gene editors and enzyme-replacement therapies). Major bottlenecks, including endosomal entrapment, inefficient endosomal escape, and suboptimal direct translocation efficiency, are increasingly being addressed.

One of the next frontiers certainly lies in achieving cell specificity: while many strategies exploit positive charges to promote interactions with negatively charged cell membranes, such interactions often yield nonspecific binding. Activatable systems, which are responsive to cues such as the tumor microenvironment, offer promising solutions.^259^

Taken together, chemically driven protein delivery offers an almost ideal research field for the next generation of researchers: first proof-of-concept studies point toward enormous potential, while still encountering significant challenges. There is no better time to join this race.

## Notes

For J.V.V.A: Institute of Advanced Energy, Kyoto University, Uji, Kyoto 611-0011, Japan
